# Identification of hub genes significantly linked to temporal lobe epilepsy and apoptosis *via* bioinformatics analysis

**DOI:** 10.3389/fnmol.2024.1300348

**Published:** 2024-02-07

**Authors:** Weiliang Wang, Yinghao Ren, Fei Xu, Xiaobin Zhang, Fengpeng Wang, Tianyu Wang, Huijuan Zhong, Xin Wang, Yi Yao

**Affiliations:** ^1^Epilepsy Center, Xiamen Humanity Hospital, Fujian Medical University, Xiamen, Fujian, China; ^2^Department of Dermatology, Xiamen Humanity Hospital, Fujian Medical University, Xiamen, Fujian, China; ^3^Department of Pharmacogenomics, College of Bioinformatics Science and Technology, Harbin Medical University, Harbin, China; ^4^Department of Neurosurgery, The First Affiliated Hospital of Harbin Medical University, Harbin, Heilongjiang, China; ^5^Department of Neurology, The First Affiliated Hospital of Harbin Medical University, Harbin, Heilongjiang, China

**Keywords:** temporal lobe epilepsy, apoptosis, bioinformatics analysis, biomarkers, classification modeling

## Abstract

**Background:**

Epilepsy stands as an intricate disorder of the central nervous system, subject to the influence of diverse risk factors and a significant genetic predisposition. Within the pathogenesis of temporal lobe epilepsy (TLE), the apoptosis of neurons and glial cells in the brain assumes pivotal importance. The identification of differentially expressed apoptosis-related genes (DEARGs) emerges as a critical imperative, providing essential guidance for informed treatment decisions.

**Methods:**

We obtained datasets related to epilepsy, specifically GSE168375 and GSE186334. Utilizing differential expression analysis, we identified a set of 249 genes exhibiting significant variations. Subsequently, through an intersection with apoptosis-related genes, we pinpointed 16 genes designated as differentially expressed apoptosis-related genes (DEARGs). These DEARGs underwent a comprehensive array of analyses, including enrichment analyses, biomarker selection, disease classification modeling, immune infiltration analysis, prediction of miRNA and transcription factors, and molecular docking analysis.

**Results:**

In the epilepsy datasets examined, we successfully identified 16 differentially expressed apoptosis-related genes (DEARGs). Subsequent validation in the external dataset GSE140393 revealed the diagnostic potential of five biomarkers (CD38, FAIM2, IL1B, PAWR, S100A8) with remarkable accuracy, exhibiting an impressive area under curve (AUC) (The overall AUC of the model constructed by the five key genes was 0.916, and the validation set was 0.722). Furthermore, a statistically significant variance (*p* < 0.05) was observed in T cell CD4 naive and eosinophil cells across different diagnostic groups. Exploring interaction networks uncovered intricate connections, including gene-miRNA interactions (164 interactions involving 148 miRNAs), gene-transcription factor (TF) interactions (22 interactions with 20 TFs), and gene-drug small molecule interactions (15 interactions involving 15 drugs). Notably, IL1B and S100A8 demonstrated interactions with specific drugs.

**Conclusion:**

In the realm of TLE, we have successfully pinpointed noteworthy differentially expressed apoptosis-related genes (DEARGs), including CD38, FAIM2, IL1B, PAWR, and S100A8. A comprehensive understanding of the implications associated with these identified genes not only opens avenues for advancing our comprehension of the underlying pathophysiology but also bears considerable potential in guiding the development of innovative diagnostic methodologies and therapeutic interventions for the effective management of epilepsy in the future.

## Introduction

Epilepsy, a chronic disorder of the central nervous system characterized by recurrent seizures without an apparent trigger and abnormal neuronal network activity, poses a significant clinical challenge (Thijs et al., 2019). While the etiology of epilepsy in many cases remains elusive, it is noteworthy that disruptions of normal brain function, stemming from various factors such as traumatic brain injury, infectious diseases, autoimmune disorders, and genetic mutations, can precipitate seizures (Pitkänen et al., 2016; Devinsky et al., 2018). With approximately 1,000 genes associated with epilepsy, mutations in these genes manifest primarily as conditions characterized by seizures (Rana and Musto, 2018).

MicroRNAs (miRNAs), as regulators of gene expression, exert multifaceted effects on epilepsy, with modulation of the inflammatory response and regulation of apoptosis identified as key mechanisms (Xie et al., 2023).

Extensive research has delved into the intricate involvement of numerous genes and signaling pathways in the development of epilepsy (Perucca et al., 2020). Recognizing the pivotal role of genetic factors in epilepsy and its treatment, ongoing efforts utilize various genetic and genomic technologies to analyze the disorder’s genetic foundations (Shevlyakov et al., 2023). High-throughput sequencing analysis of gene expression, complemented by advanced bioinformatics tools, has emerged as a cutting-edge approach for investigating disease onset and progression, offering insights into the underlying mechanisms of epilepsy.

Within the domain of challenging epileptic conditions, TLE stands out as a formidable subtype, necessitating immediate attention for comprehensive genetic diagnosis and therapeutic interventions. There exists an urgent need to refine our understanding and approach to the genetic underpinnings of TLE, with the goal of optimizing epilepsy management through non-surgical treatment modalities.

In this study, we employed bioinformatics methodologies to identify differentially expressed apoptosis-related genes (DEARGs) in patients with TLE compared to normal individuals. Our investigation encompassed the exploration of prospective biomarker candidates and the development of disease diagnostic models. Furthermore, we conducted enrichment analyses and constructed gene networks based on the finalized DEARGs. To unveil potential options for epilepsy treatment, we enhanced drug molecular docking, contributing to the advancement of therapeutic possibilities.

## Materials and methods

### Data source

**FIGURE 1:**
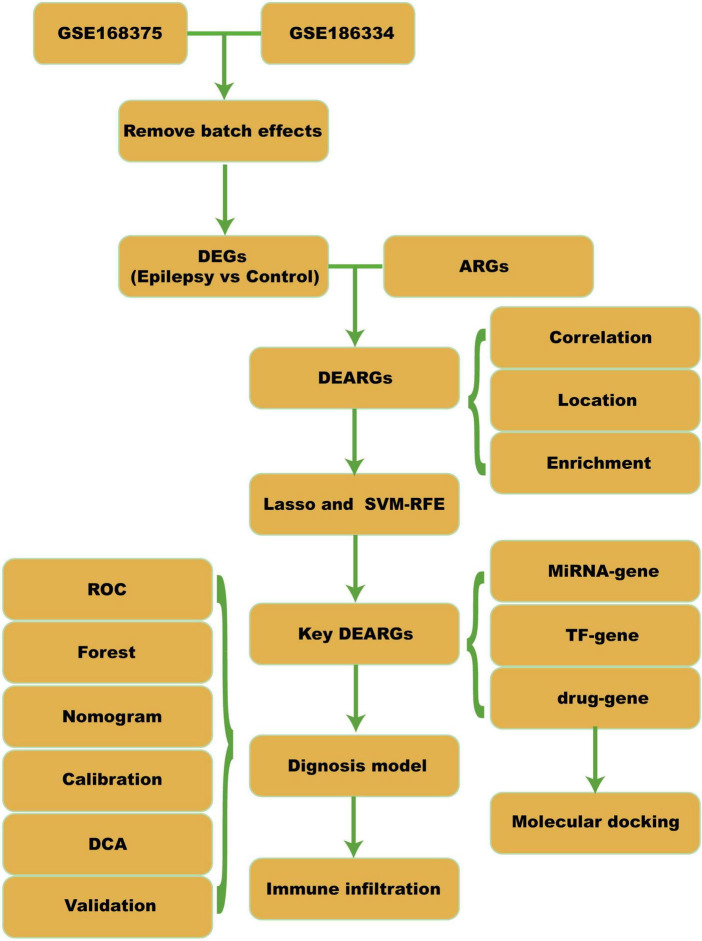
Workflow. DEGs, differential expressed genes; ARGs, apoptosis-related genes; DEARGs, differential expressed apoptosis-related genes; SVM-RFE, support vector machine-recursive feature elimination; TF, transcription factor; ROC, receiver operating curve; DCA, decision curve analysis.

As depicted in Figure 1, the comprehensive workflow adopted in this study is outlined. We downloaded epilepsy data from the gene expression omnibus (GEO) (Barrett et al., 2012) (Supplementary Table 1). GSE168375 includes 31 epilepsy patient brain tissue samples and 12 normal brain tissue samples as controls (Krawczyk et al., 2022). GSE186334 includes 24 epilepsy patient cortical samples and 12 healthy control cortical samples (Gomes-Duarte et al., 2022). GSE140393 validation set consists of 12 epilepsy patient cortical samples and 9 healthy control cortical samples (Pai et al., 2022). Additionally, we downloaded a set of 580 genes associated with apoptosis from PMID36341760 (Zou et al., 2022) (Supplementary Table 2).

GSE168375 Dataset: Control Group: 12 samples, average age 35.25 (3 males, 9 females). Epilepsy Group: 31 samples (1 missing gender/age), average age 6.7 (13 males, 17 females).

GSE186334 Dataset: Epilepsy Group: 10 samples with available clinical information. Average age for patients E5, E6, E7: 56.667 (2 females, 1 male).

GSE140393 Dataset: Control Group: 9 samples from 3 patients, average age 35.3 (2 males, 1 female). Epilepsy Group: 21 samples from 5 patients, average age 24.8 (4 males, 1 female).

### Differential expression analysis

We used the “sva” package (v3.40.0) in R to perform batch correction on the GSE168375 and GSE186334 datasets (Leek et al., 2012). Subsequently, principal component analysis (PCA) was conducted. Differential analysis was performed on the integrated epilepsy dataset using the “limma” package (v3.48.3) to identify DEGs between the epilepsy and control groups (Ritchie et al., 2015). Genes with logFC (fold change, FC) > 0.58 and *p*-value < 0.05 were considered upregulated DEGs, while genes with logFC < −0.58 and *p*-value < 0.05 were considered downregulated DEGs. The correlation analysis of DEARGs was performed using the “psych” package (v2.3.6) in R to calculate the Spearman correlation between gene expressions. Gene positions on chromosomes were obtained using the “biomaRt” package (v2.54.1) and visualized using the genes package (v0.5.0) (Kasprzyk, 2011).

### Enrichment analysis

The “clusterProfiler” package (v4.7.1.3) is used for performing gene ontology (GO) and Kyoto Encyclopedia of Genes and Genomes (KEGG) pathway enrichment analyses (Yu et al., 2012). The enrichment results are visualized using the “GOplot” package (v1.0.2) and “enrichplot” package (v1.18.4) (Walter et al., 2015; Wu et al., 2021). The “pathview” package (v1.40.0) in R is used for visualizing the KEGG pathways (Luo and Brouwer, 2013). Gene set enrichment analysis (GSEA) is a computational method used to determine whether a predefined set of genes shows statistically significant differences between two biological states (Subramanian et al., 2005). We downloaded the reference gene set “c2.cp.kegg.v7.4.entrez.gmt” from the MSigDB database (Liberzon et al., 2015). The “clusterProfiler” package (v4.0.5) includes GSEA methods for conducting enrichment analysis and visualization of the dataset (Wu et al., 2021). Gene set variation analysis (GSVA) is used to analyze the variation of gene sets (Subramanian et al., 2005). We obtained the reference gene set “h.all.v7.4.symbols.gmt” from the MSigDB database to perform GSVA analysis on different groups of the integrated GEO dataset. The “GSVA” package (Hanzelmann et al., 2013) (v1.40.0) converts the expression matrix into a pathway enrichment score matrix and uses the “lmFit” function from the “limma” package (v3.48.0) to identify different pathways and calculate *p*-values (Ritchie et al., 2015).

### Identification of optimal diagnostic gene biomarkers for epilepsy

We used the Lasso regression method with the glmnet R package (v4.1-2) to screen all DEARGs (Friedman et al., 2010). Then, we employed the SVM-RFE algorithm with the caret R package (v6.0.94) to further select features associated with epilepsy-related apoptosis genes. The intersection of the features selected by Lasso and SVM-RFE was considered as the set of genes for constructing the diagnostic model. We performed a Friends analysis using the R package GOSemSim (v2.24.0) (Yu, 2020). Using logistic regression based on the selected feature genes, we constructed the diagnostic model. The diagnostic score was calculated using the gene expression levels and the coefficients obtained from the multiple regression model as follows:


$$\text{diagnosis}Score=\sum_{i}Coefficient(hubgene_{i})*$$

$$mRNAExpression(hubgene_{i})$$

Furthermore, we divided the combined epilepsy dataset into a high-score diagnostic group and a low-score diagnostic group. We used the “pROC” R package (v1.18.0) to plot the ROC curve for the diagnostic model and epilepsy status (Robin et al., 2011). The calibration plot was generated using the rms R package (v6.2-0) to assess the accuracy and discriminative ability of the diagnostic model. Finally, the decision curve analysis (DCA) plot was created using the “ggDCA” R package (v1.1) to evaluate the accuracy and discriminative ability of the logistic regression model (Vickers and Elkin, 2006).

### Immune infiltration analysis

We uploaded the expression profile data of the epilepsy dataset to the CIBERSORTx website and used the provided LM22 gene set to calculate the abundance of 22 immune cell types in each patient (Wu et al., 2021). The correlation between genes related to epilepsy in different groups of immune cells was calculated using the Spearman algorithm, and the correlation heatmap was generated using the R package ggplot2 (v3.3.6).

### Gene network construction

We conducted further predictive research on the diagnostic model genes using the miRNet database (Chang et al., 2020). We downloaded information on small-molecule drug targets from DrugBank and used this information to predict small-molecule drugs that could interact with the diagnostic model genes (Wishart et al., 2008). We utilized Cytoscape (v3.8.2) for visualization and network analysis (Shannon et al., 2003).

### Molecular docking analysis

We downloaded the 2D or 3D structures of small molecule drugs from the PubChem database and used Chem3D software to convert the 2D structures into 3D structures (Kim et al., 2021; Bateman et al., 2023). Next, we searched the UniProt and PDB databases for human receptor protein structures corresponding to the genes of interest (Burley et al., 2017). With PyMOL software, we visualized the protein structures (Seeliger and de Groot, 2010). Finally, we used Autodock Vina software to identify the active binding sites involved in the interaction between the ligands and receptors (Seeliger and de Groot, 2010).

### Statistical analysis

All data calculations and statistical analyses were conducted utilizing the R programming language (version 4.2.3). For multiple testing corrections, we utilized the Benjamini–Hochberg method with false discovery rate adjustment. For comparisons between two groups of continuous variables, we estimated the statistical significance of normally distributed variables using an independent Student’s *t*-test. Differences in non-normally distributed variables were analyzed with the Wilcoxon test. We used Spearman correlation analysis to calculate correlation coefficients between different molecules. All *p*-values were two-sided, and statistical significance was defined as *p* < 0.05.

## Results

### Batch correction and DEARG analysis in epilepsy vs. control groups

**FIGURE 2:**
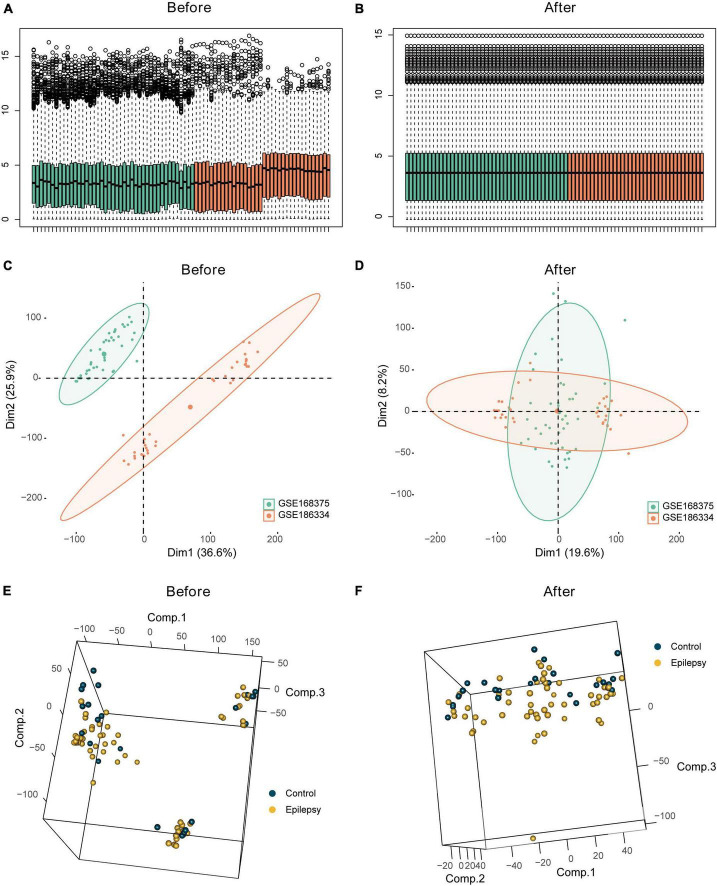
Batch effect analysis of the integrated epilepsy dataset. Box plots depicting combined epilepsy data pre- **(A)** and post- **(B)** batch correction. Batch analysis of combined epilepsy data pre- **(C)** and post- **(D)** batch correction employing PCA. Employment of PCA for differential analysis of epilepsy and control groups pre- **(E)** and post- **(F)** batch correction in the integrated epilepsy dataset.

GSE168375 and GSE186334 underwent batch correction, and the pre- and post-batch effect removal data sets were compared using a distribution boxplot and PCA plot (Figure 2).

**FIGURE 3:**
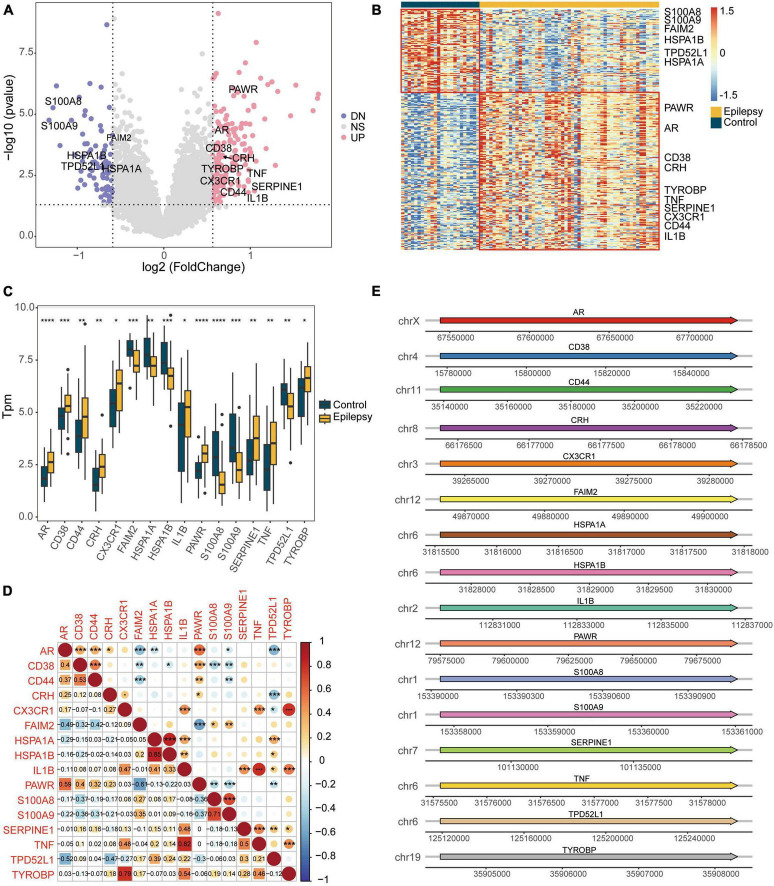
Differential expression analysis of the integrated dataset between the epilepsy group and the control group. **(A)** Volcano plot of the DEGs. **(B)** Heatmap of the DEGs. **(C)** Box plot showing the expression of apoptotic genes that are differentially expressed. **(D)** Spearman correlation heatmap showing the correlation of DEARGs. **(E)** Diagram showing the positions of DEARGs on the chromosomes. *P < 0.05, **P < 0.01, ***P < 0.001, ****P < 0.0001. UP, up-regulated; DN, down-regulated; NS, not significant.

We intersected the DEGs between the epilepsy and control groups with apoptosis-related genes, resulting in 16 DEARGs, 10 of which were upregulated and 6 were downregulated. Figure 3A displays a volcano plot of the DEGs, Figure 3B depicts a heatmap of the DEGs, and Figure 3C shows boxplots of the DEARGs. The volcano plot, heatmap, and boxplots illustrate the expression patterns of genes, including S100A8 and S100A9, which experienced downregulation in the epilepsy group, whereas PAWR, AR, CD38, and others were upregulated. The Spearman correlation coefficients were calculated amongst the DEARGs (Figure 3D). It was discovered that the expression of HSPA1B and HSPA1A exhibited the highest correlation (*r* = 0.85, *p* < 2e-16), while the expression of IL1B and TNF showed positive correlation (*r* = 0.82, *p* < 2e-16). Figure 3E illustrates the chromosomal positions of the 16 DEARGs. The figure indicates that AR is situated on the X chromosome whereas S100A8 and S100A9 are located on chromosome 1.

### Functional pathway analysis and enrichment in epilepsy group

**FIGURE 4:**
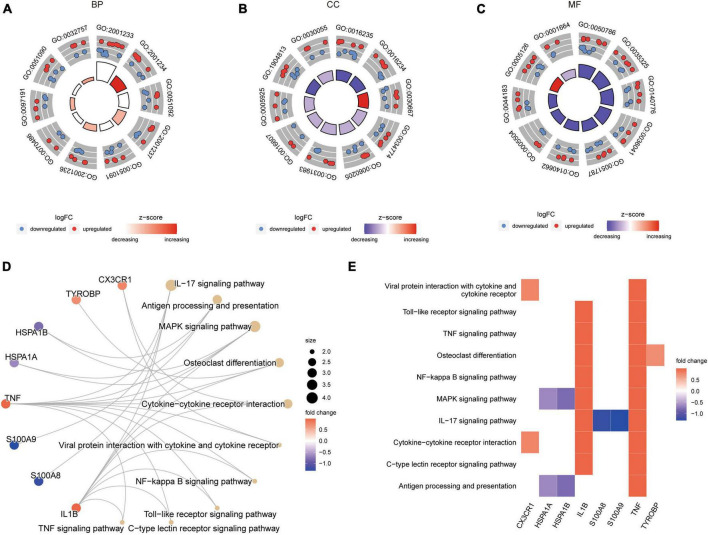
GO and KEGG enrichment results of DEARGs between the epilepsy group and the control group. **(A–C)** Enriched GO pathways associated with Biological Process **(A)**, Cell Component **(B)** and Molecular Function **(C)**. LogFC represents the log_2_ (fold change) of gene expression between epilepsy group and control group. **(D)** Enriched KEGG pathways. Dot size represents the number of genes in the pathway. **(E)** Heat map of enriched KEGG pathways.

The results (Supplementary Table 3) revealed that in the epilepsy group, the enriched GO pathways were primarily associated with BP, including GO:2001233 (regulation of apoptotic signaling pathway), GO:0051092 (positive regulation of NF-kappaB transcription factor activity), GO:2001237 (negative regulation of extrinsic apoptotic signaling pathway), among others (Figure 4A). In terms of CC, the enriched GO terms included GO:0016235 (aggresome), GO:0016234 (inclusion body), GO:0030667 (secretory granule membrane), and others (Figure 4B). For MF, the enriched GO terms included GO:0050786 (RAGE receptor binding), GO:0035325 (Toll-like receptor binding), GO:0140776 (protein-containing complex destabilizing activity), and others (Figure 4C). Figure 4D illustrated the enriched KEGG pathways, mainly involving the IL-17 signaling pathway, Antigen processing and presentation, and others. Figure 4E presented a heat map of the enriched KEGG pathways, providing a visual representation of the pathway-gene associations (Supplementary Table 4).

Supplementary Figure 1A illustrated the role of genes in the TNF signaling pathway. In this pathway, the inflammatory factors TNF and IL1B activate TNFR1 and TNFR2, respectively, leading to the activation of downstream pathways that can induce cell apoptosis or perform other functions. Supplementary Figure 1B depicted the specific details of the Antigen processing and presentation pathway. TNF activation triggered a series of subsequent reactions, including the involvement of PA28, which assisted in the functioning of CD8 T cells, NK cells, CD4 T cells, and other immune cells.

**FIGURE 5:**
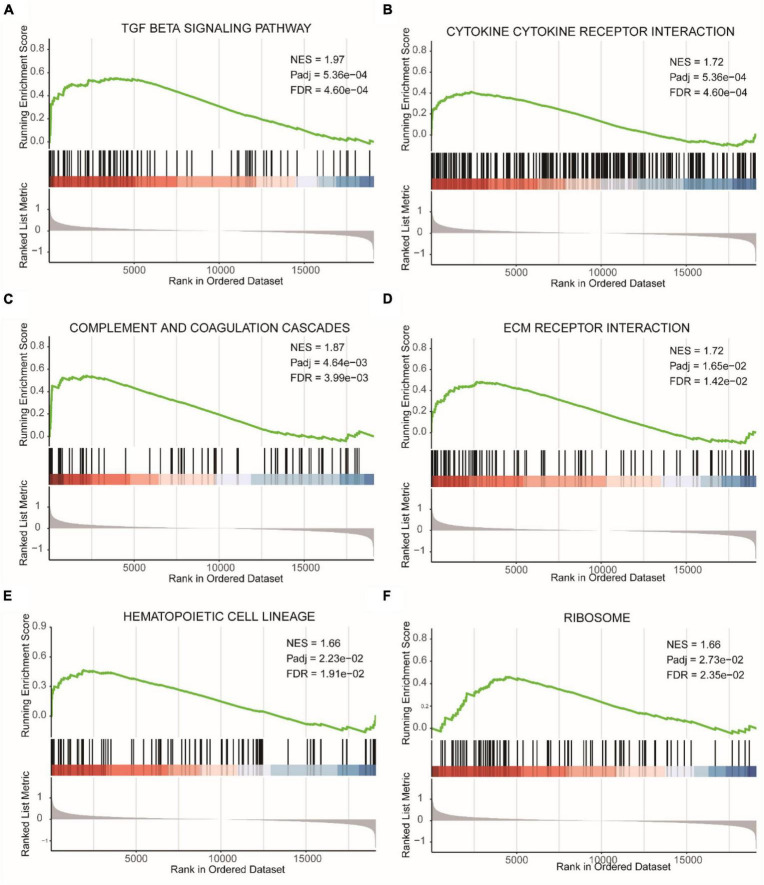
GSEA results of epilepsy group and the control group. **(A)** TGF beta signaling pathway. **(B)** Cytokine cytokine receptor interaction. **(C)** Complement and coagulation cascades. **(D)** ECM receptor interaction. **(E)** Hematopoietic cell lineage. **(F)** Ribosome. NES, Normalized Enrichment Score. Pathways with FDR (False Discovery Rate) < 0.05 are generally considered to be statistically significant.

Due to the subjective nature of selecting threshold values for DEGs, we also performed GSEA based on gene expression fold changes (Supplementary Table 5). Figure 5A represented the TGF BETA SIGNALING PATHWAY, Figure 5B showed CYTOKINE CYTOKINE RECEPTOR INTERACTION, Figure 5C displayed COMPLEMENT AND COAGULATION CASCADES, Figure 5D illustrated ECM RECEPTOR INTERACTION, Figure 5E depicted HEMATOPOIETIC CELL LINEAGE, and Figure 5F represented RIBOSOME. All of these pathways were enriched in the epilepsy group.

We conducted GSVA based on gene expression profiles (Supplementary Table 6). Supplementary Figure 2A displayed stronger functional enrichment of TGF_BETA_SIGNALING, KRAS_SIGNALING_UP, and EPITHELIAL_MESENCHYMAL_TRANSITION in the epilepsy group, while REACTIVE_OXYGEN_SPECIES_PATHWAY, KRAS_SIGNALING_DN, and HEME_METABOLISM showed weaker functional enrichment in the epilepsy group. We examined the Spearman correlation between the gene with the highest absolute fold change in expression between the two groups (S100A9) and functional pathways. Supplementary Figure 2B presented a scatter plot showing the correlation between the GSVA score of TGF_BETA_SIGNALING and S100A9 gene expression, which exhibited a negative correlation (*r* = −0.36, *p* = 0.0013). Supplementary Figure 2C demonstrated a positive correlation between the GSVA score of REACTIVE_OXYGEN_SPECIES_PATHWAY and S100A9 gene expression (*r* = 0.35, *p* = 0.0016). Finally, Supplementary Figure 2D showed a negative correlation between the GSVA score of EPITHELIAL_MESENCHYMAL_TRANSITION and S100A9 gene expression (*r* = −0.32, *p* = 0.0041).

### Diagnostic model and validation analysis

**FIGURE 6:**
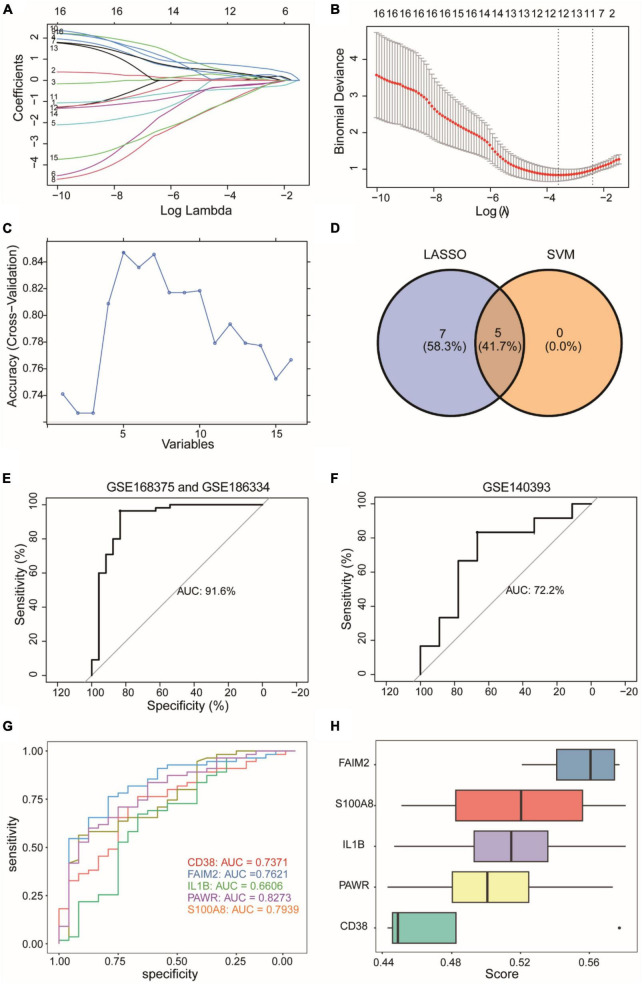
Construction of a diagnostic model for an integrated epilepsy dataset. **(A,B)** Selection of twelve feature genes from sixteen DEARGs using Lasso algorithm. **(C)** Selection of five feature genes from sixteen DEARGs using SVM-RFE algorithm. SVM-RFE: Support Vector Machine - Recursive Feature Elimination. **(D)** Five feature genes determined by the combined results of both algorithms. **(E)** Diagnostic model performance with identified biomarkers. **(F)** External validation using GSE140393 dataset. **(G)** Individual gene ROC curves. ROC, Receiver Operating Characteristic Curve. **(H)** Functional importance of diagnostic genes. The horizontal coordinate (score) represents similarity score of a gene with other genes based on semantic similarity. The higher the score, the higher the correlation with other genes, and the more important the gene.

We employed the Lasso algorithm to select twelve feature genes from sixteen DEARGs (Figures 6A, B). Furthermore, we used the SVM-RFE algorithm to select five genes from the sixteen DEARGs (Figure 6C). Combining the results of both algorithms, we identified five feature genes (CD38, FAIM2, IL1B, PAWR, S100A8) as diagnostic biomarkers for epilepsy grouping (Figure 6D). Using logistic regression, a diagnostic model for epilepsy grouping was constructed with these five key genes. The risk score was calculated as: risk score = 0.596134*exp(CD38)–1.312078*exp(FAIM2) + 0.612583*exp(IL1B) + 1.568740*exp(PAWR)–0.687394*exp(S100A8). The constructed diagnostic model demonstrated high accuracy in diagnosing epilepsy, with an area under curve (AUC) value of 0.916 as shown in Figure 6E.

We used GSE140393 as a validation set and calculated the ROC curve accordingly (Figure 6F). The results showed that the diagnostic model had high accuracy in diagnosing epilepsy in the external validation dataset GSE140393, with an AUC of 0.722. Furthermore, individual ROC curves were generated for each gene separately (Figure 6G). The AUC values ranged from 0.66 to 0.82, indicating that the diagnostic capacity of models based on individual genes was lower than that of the overall 5-gene model. Lastly, we conducted GO analysis to assess the functional importance of the 5 diagnostic genes using Friends analysis (Figure 6H). The results showed that the gene FAIM2 played an important functional role and might be a crucial gene in the context of epilepsy.

**FIGURE 7:**
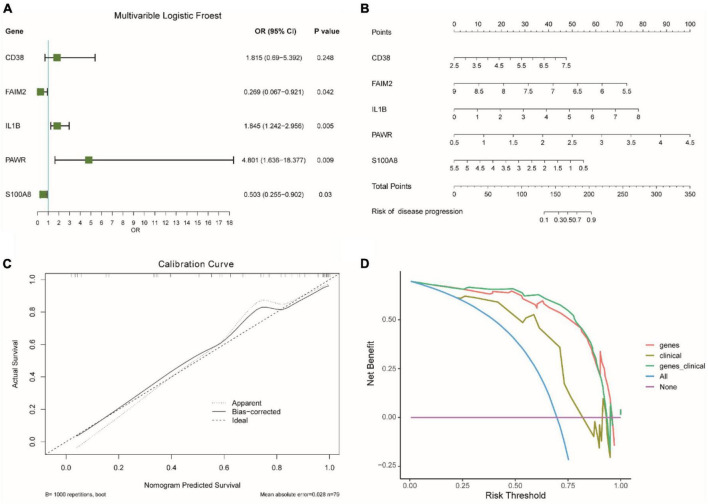
Evaluation of diagnostic models for integrated epilepsy datasets. **(A)** Impact analysis of genes on disease. OR: Odds Ratio. OR < 1 means that the gene is a protective factor, OR > 1 means that the gene is a risk factor. CI, confidence interval. **(B)** Nomogram analysis of diagnostic model. **(C)** Calibration curve for diagnostic model accuracy. **(D)** Decision curve for clinical effectiveness.

We analyzed the impact of each gene on the disease and visualized it as a forest plot (Figure 7A). The results showed that IL1B and PAWR were risk factors. On the other hand, FAIM2 and S100A8 were protective factors. The diagnostic capability of the diagnostic model was assessed using Nomogram analysis (Figure 7B). It was found that PAWR made a significant contribution to the diagnosis of epilepsy. The calibration curve (Figure 7C) is primarily used to assess the accuracy of the diagnostic model. The fitted curve showed a high degree of overlap with the reference curve (dashed line), indicating good predictive performance of the diagnostic model. The decision curve (Figure 7D) represented the net benefit against threshold probabilities. This indicates that the clinical effectiveness of the model was better within this range. Upon observation, it can be concluded that the diagnostic model showed good clinical effectiveness.

We divided the samples into a high-risk diagnostic group and a low-risk diagnostic group based on the median risk score from the diagnostic model and calculated the expression differences between the two groups. Then, we performed GSEA analysis on the high-risk and low-risk diagnostic groups based on the fold changes in gene expression.

**FIGURE 8:**
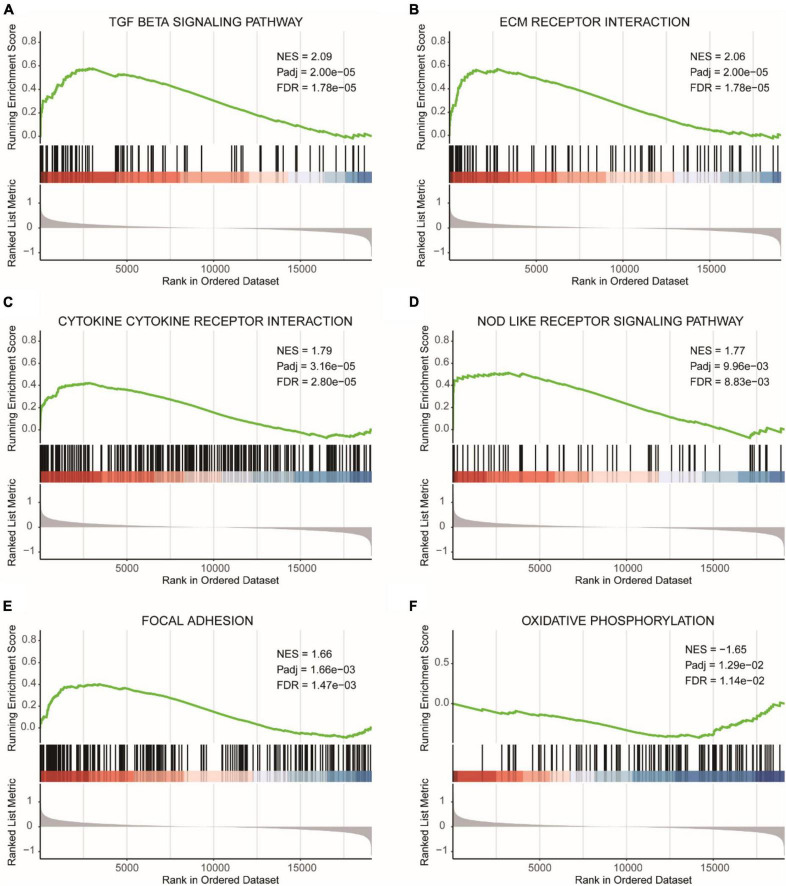
GSEA results for high and low-risk diagnostic groups. **(A)** GSEA analysis results for the TGF beta signaling pathway. **(B)** GSEA analysis results for ECM receptor interaction. **(C)** GSEA analysis results for cytokine cytokine receptor interaction. **(D)** GSEA analysis results for NOD like receptor signaling pathway. **(E)** GSEA analysis results for focal adhesion. **(F)** GSEA analysis results for oxidative phosphorylation. NES, normalized enrichment score. Pathways with FDR (False Discovery Rate) < 0.05 are generally considered to be statistically significant.

Figure 8A showed the GSEA analysis results for the TGF BETA SIGNALING PATHWAY, Figure 8B displayed the GSEA analysis results for ECM RECEPTOR INTERACTION, Figure 8C presented the GSEA analysis results for CYTOKINE CYTOKINE RECEPTOR INTERACTION, Figure 8D illustrated the GSEA analysis results for NOD LIKE RECEPTOR SIGNALING PATHWAY, and Figure 8E represented the GSEA analysis results for FOCAL ADHESION. All five pathways were found to be more enriched in the high-risk diagnostic group. Figure 8F showed the GSEA analysis results for OXIDATIVE PHOSPHORYLATION, which was more enriched in the low-risk diagnostic group.

We performed GSVA analysis on the gene expression profiles. Supplementary Figure 3A presented the results of the functional enrichment analysis, which showed that UV_RESPONSE_DN, TGF_BETA_SIGNALING, KRAS_SIGNALING_UP, and EPITHELIAL_MESENCHYMAL_TRANSITION were functionally enriched to a greater extent in the high-risk diagnostic group, while REACTIVE_OXYGEN_SPECIES_PATHWAY, MYC_TARGETS_V2, KRAS_SIGNALING_DN, and GLYCOLYSIS were more functionally enriched in the low-risk diagnostic group.

Subsequently, we examined the Spearman correlation between the risk diagnostic score and functional pathways. Supplementary Figure 3B showed the scatter plot of the correlation between the GSVA score of GLYCOLYSIS and the risk diagnostic score, revealing a negative correlation (*r* = −0.3, *p* = 8.1e-03). Supplementary Figure 3C displayed the negative correlation between the GSVA score of MYC_TARGETS_V2 and the risk diagnostic score (*r* = −0.39, *p* = 4.5e-04). Supplementary Figure 3D demonstrated the negative correlation between the GSVA score of REACTIVE_OXYGEN_SPECIES_PATHWAY and the risk diagnostic score (*r* = −0.45, *p* = 4.2e-05). Supplementary Figure 3E represented the positive correlation between the GSVA score of TGF_BETA_SIGNALING and the risk diagnostic score (*r* = −0.41, *p* = 2.1e-04). Supplementary Figure 3F illustrated the negative correlation between the GSVA score of EPITHELIAL_MESENCHYMAL_TRANSITION and the risk diagnostic score (*r* = 0.35, *p* = 1.6e-03).

### Differential immune cell infiltration and correlation analysis

**FIGURE 9:**
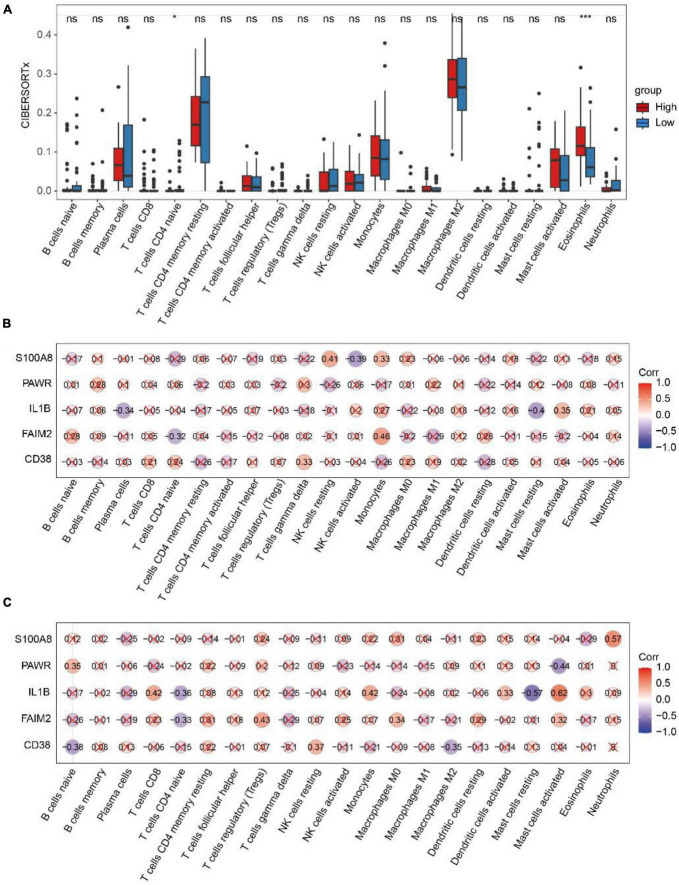
Analysis of CIBERSORTx immune infiltration. **(A)** The difference of immune cell infiltration with CIBERSORTx method in the high and low risk diagnosis group. * represents *p*-value < 0.05, ** represents *p*-value < 0.01, *** represents *p*-value < 0.001, **** represents *p*-value < 0.0001, ns represents not significant. **(B)** Spearman correlation between diagnostic genes and immune cell infiltration in the high-risk group. **(C)** Spearman correlation between diagnostic genes and immune cell infiltration in the low-risk group. Red marks indicate *p*-value > 0.05.

We compared the differences in immune cell infiltration abundance between the high and low-risk diagnostic groups for each of the 22 immune cell types (Figure 9A). The results showed significant differences in the infiltration abundance of T cells CD4 naive and Eosinophils. T cells CD4 naive had higher immune infiltration in the low-risk diagnostic group, while Eosinophils had higher immune infiltration in the high-risk diagnostic group.

In the high-risk group (Figure 9B), FAIM2 and Monocyte showed positive correlations (*r* = 0.46, *p* = 2.6e-02), while they exhibited negative correlation with T cells CD4 naive (*r* = −0.32, *p* = 4.7e-02). In the low-risk group (Figure 9C), FAIM2 showed a negative correlation with T cells CD4 naive (*r* = −0.33, *p* = 3.8e-02), and IL1B showed a negative correlation with T cells CD4 naive (*r* = −0.36, *p* = 2.3e-02).

**FIGURE 10:**
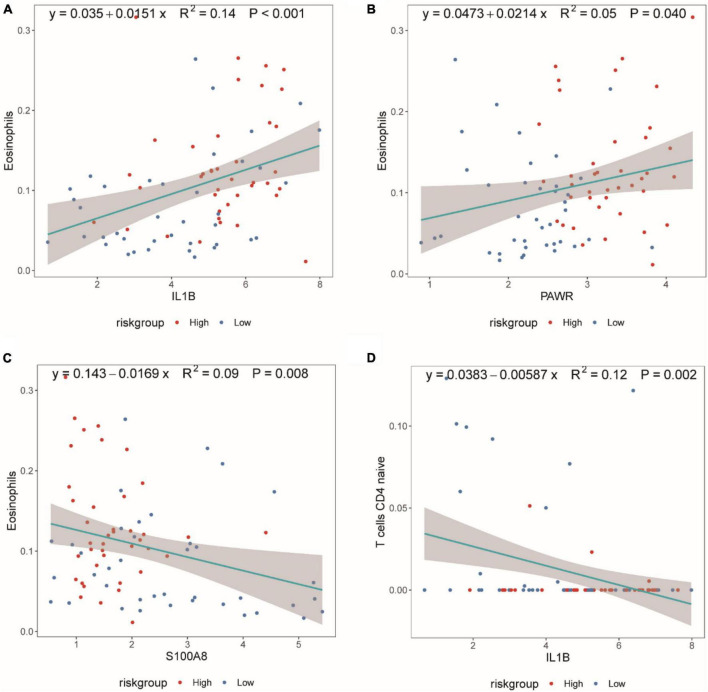
Scatter plot of the Spearman correlation between CIBERSORTx immune infiltration results and diagnostic genes. **(A)** Spearman correlation between IL1B expression and eosinophil proportion. **(B)** Spearman correlation between PAWR expression and eosinophil proportion. **(C)** Spearman correlation between S100A8 expression and eosinophil proportion. **(D)** Spearman correlation between IL1B expression and T cells CD4 naive proportion. The correlation index, R2, represents the degree of fit of the regression equation, with values between [0,1].

Figure 10A demonstrated that as the expression of IL1B increased, the proportion of Eosinophils also increased. Figure 10B illustrated that with an increase in PWAR expression, the proportion of Eosinophils also increased. Figure 10C showed that as S100A8 expression increased, the proportion of Eosinophils decreased. Figure 10D displayed that as IL1B expression increased, the proportion of T cells CD4 naive decreased.

### Integrated network analysis of gene-miRNA, gene-TFs, and gene-drug interactions

**FIGURE 11:**
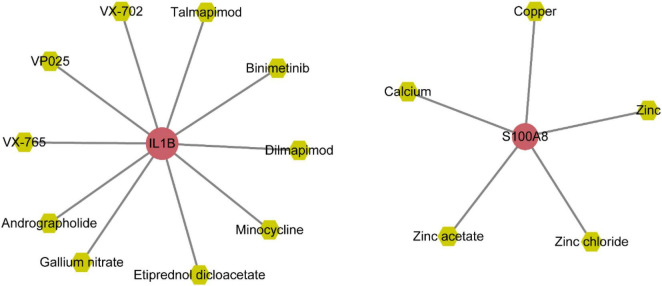
Network of diagnostic genes and small molecule drugs.

The gene-miRNAs network consisted of 164 interaction pairs involving 148 miRNAs (Supplementary Figure 4A and Supplementary Table 8). The gene-TFs network consisted of 22 interaction pairs involving 20 TFs (Supplementary Figure 4B and Supplementary Table 7). The gene-drug small molecule network consisted of 15 interaction pairs involving 15 small molecule drugs (Figure 11 and Supplementary Table 9).

### Molecular docking analysis of IL1B with small molecules: insights into interaction mechanisms

According to the gene-small molecule interaction data, it was determined that two out of the five selected feature genes, IL1B and S100A8, interacted with the small molecules. Following the selection criteria for protein structures (presence of at least one ligand, lower resolution preferred), we identified the 5R8Q structure of IL1B that met the requirements. Subsequently, we performed molecular docking analysis between IL1B (5R8Q) and the small molecules Andrographolide, Binimetinib, Dilmapimod, Etiprednol Dicloacetate, and others.

**FIGURE 12:**
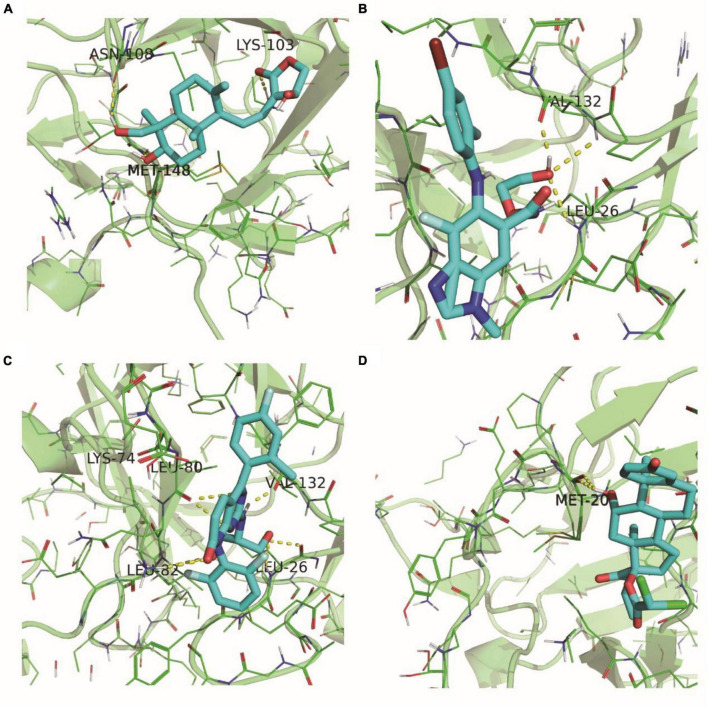
5R8Q configuration of IL1B and docking results of drug molecules. IL1B’s 5R8Q conformation with Andrographolide **(A)**, Binimetinib **(B)**, Dilmapimod **(C)** and Etiprednol Dicloacetate **(D)**.

Figure 12A depicted the binding scenario between IL1B and Andrographolide, where hydrogen bonds with residues LYS-103, MET-148, and ASN-108 were observed, and the predicted binding affinity was −7.2. In Figure 12B, the binding interaction between IL1B and Binimetinib was shown, with a hydrogen bond formed with residue LEU-26, and the predicted binding affinity was −7.2. Figure 12C illustrated the binding of IL1B and Dilmapimod, with hydrogen bonds formed with residues LYS-74 and VAL-163, and the predicted binding affinity was −9.0. Figure 12D displayed the binding between IL1B and IL1B_Etiprednol Dicloacetate, forming a hydrogen bond with residue MET-20, and the predicted binding affinity was −6.1. Meanwhile, we also show the spatial structure of the 5R8Q construct of IL1B (Supplementary Figure 5).

## Discussion

Epilepsy stands out as a prevalent comorbidity in neurodegenerative diseases, and alterations in neuronal proteins present in cerebrospinal fluid and blood hold promise as potential biomarkers for primary lesions within the central nervous system. Investigating the mechanisms and clinical implications of these neuronal biomarkers shared between epilepsy and neurodegenerative diseases is crucial for advancing diagnostic and therapeutic strategies (Negi et al., 2023). Given the diverse nature of epilepsy, distinct biomarkers characterize each condition. The identification of patient-specific biomarker profiles can contribute to personalized epilepsy treatment, facilitate the monitoring of antiepileptic interventions, and aid in the identification of candidates suitable for surgical interventions (Kobylarek et al., 2019).

Apoptosis of neurons and glial cells is important in the pathogenesis of epilepsy, especially TLE. The study of the mechanisms of apoptosis is crucial for the creation of a new generation of neuroprotective and antiepileptic drugs that can play an effective role, especially in the case of TLE (Teocchi and D’Souza-Li, 2016). Ultrastructural and immunohistochemical signs of apoptosis were found in temporal lobe neurons and oligodendrocytes from patients with TLE. Pro-inflammatory cytokines (TNF-α, NF-kB) associated with apoptosis were elevated. It was shown that apoptosis of oligodendrocytes plays an important role in the etiology of TLE (Bazhanova and Kozlov, 2022). It has been reported that neuroinflammatory processes occur in epileptic foci and lead to apoptosis through exogenous receptor and mitochondrial pathways, with the exogenous pathway predominating. Expression of pro-apoptotic proteins has also been observed in the perifocal region. Thus, active neuroinflammation in temporal lobe epileptic foci and perifocal regions, as well as an imbalance in the anti-apoptotic system in the perifocal region, promotes further degradation of the hyperexcitable foci and progression of epileptic encephalopathy (Litovchenko et al., 2021).

In our study, a total of 249 differentially expressed genes (DEGs) were identified, with 163 upregulated and 86 downregulated. Further analysis focused on DEGs common to both the epilepsy and control groups, intersecting them with genes associated with apoptosis, leading to the discovery of 16 differentially expressed apoptosis-related genes (DEARGs). Subsequent functional analyses, including gene ontology (GO), Kyoto Encyclopedia of Genes and Genomes (KEGG), gene set enrichment analysis (GSEA), and gene set variation analysis (GSVA), revealed enrichment in crucial biological processes and signaling pathways, such as the regulation of apoptotic signaling, positive regulation of NF-κB transcription factor activity, IL-17 signaling, and antigen processing and presentation. These pathways play pivotal roles in maintaining cellular functions and regulatory mechanisms, with potential implications for therapeutic interventions (Singh and Singh, 2020).

Five feature genes, namely CD38, FAIM2, IL1B, PAWR, and S100A8, were identified as diagnostic biomarkers for epilepsy subtyping, forming the basis for constructing a disease classification model. Notably, CD38, a transmembrane glycoprotein, emerged as a key player in epilepsy pathogenesis due to its involvement in NAD+ degradation and intracellular calcium ion balance (Guerreiro et al., 2020; Khodaverdian et al., 2021). Targeting CD38 presents a potential therapeutic avenue. FAIM2, a neuron-specific protein, exhibited neuroprotective effects, making it a plausible target for therapeutic interventions (Reich et al., 2011). IL-1β, a potent pro-convulsant, demonstrated elevated expression in epilepsy patients, indicating its potential as a therapeutic target (Santos et al., 2021). One study found a decrease in IL-1β and IL-6 levels in patients with TLE after surgical treatment, at which point seizures disappeared in 70% of patients. Follow-up studies confirmed that inflammatory changes disappeared 1 year after surgery, seizures disappeared in the majority of patients, and that the process of apoptotic death was associated with inflammation (Lorigados Pedre et al., 2018). PAWR, implicated in various cancers, and S100A8, a regulator of inflammatory responses, further expanded the repertoire of potential therapeutic targets (Rah et al., 2015; Pruenster et al., 2016; Tan et al., 2020; He et al., 2021). Many studies have demonstrated increased expression of the protective S100 protein and the pro-apoptotic protein caspase-3 in brain tissue in epileptic focal regions, and these proteins are thought to play a role in the etiology of epilepsy (Sitovskaya et al., 2023).

Our diagnostic model, categorizing samples into high-risk and low-risk groups, revealed distinct immune infiltrations. Notably, the low-risk group exhibited higher levels of T cell CD4 naive immune infiltration, while the high-risk group displayed elevated eosinophil levels. These findings suggest the involvement of pro-inflammatory and anti-inflammatory CD4+ T cell subsets and implicate eosinophils in the neurotoxic effects contributing to seizures. Collectively, the DEARGs appear to modulate neuroinflammation and the immune microenvironment, providing insights into potential therapeutic avenues (Durack et al., 1979; Vannucci et al., 2001; De Francesco et al., 2021).

Exploring the interactions of genes with other biomolecules, including miRNAs and TFs, revealed significant possibilities for small-molecule drug targets. Leveraging the miRNet database, we identified miRNAs and TFs associated with the diagnostic genes. Molecular docking analyses, considering the relationships between diagnostic genes and small molecule drugs, highlighted the 5R8Q conformation of IL1B as a potential target. Among the tested drugs, Andrographolide, known for its antiepileptic activity, exhibited promising interactions with IL1B, suggesting its potential as a therapeutic option for epilepsy (Huang et al., 2022).

Previous studies have found that epilepsy is closely related to apoptosis, but the mechanisms involved are not yet fully understood (Sokolova et al., 2022). The current study speculates that the mechanism may be closely related to the development of neuroinflammation and the elevation of pro-inflammatory factors (TNF-α, NF-kB, IL-1β and IL-6) (Fuller et al., 2020; Sah et al., 2021; Yamanaka et al., 2021). Its conclusion was further confirmed in our current bioinformatics analysis study. And, it was also confirmed by the decrease in IL-1β and IL-6 and seizure control in TLE patients after surgery (Lorigados Pedre et al., 2018). In addition, we identified five genes that may serve as diagnostic and therapeutic targets for TLE, of which IL1B and S100A8 are consistent with previous studies (Lorigados Pedre et al., 2018; Sitovskaya et al., 2023), and the other three genes (CD38, FAIM2 and PAWR) are our innovative findings.

We acknowledge the possibility of false positive findings in our study, given certain limitations in the dataset we utilized. In addressing this concern, we conducted an extensive review of relevant literature, as mentioned earlier, which provides some support for our conclusions.

Considering the urgency of advancing our understanding of epilepsy treatment, we are eager to share our findings with the scientific community promptly. Nevertheless, we are fully aware of the partial nature of our conclusions and the potential for false positives. To address these limitations comprehensively, we plan to undertake experimental validation in the future. This additional step aims to enhance the robustness of our discoveries.

In summary, our research further confirms the association between epilepsy and apoptosis. We have successfully identified noteworthy differentially expressed apoptosis-related genes, including CD38, FAIM2, IL1B, PAWR, and S100A8. These genes exhibit diagnostic potential for TLE with a noteworthy accuracy (AUC = 0.916). Furthermore, our findings aim to provide novel insights into the treatment of TLE. Through drug-molecule docking analysis, we have discovered the therapeutic potential of Andrographolide in treating epilepsy.

The limitations of our study are primarily associated with the data utilized. Due to the adoption of different datasets, certain clinical information within the datasets is not clearly defined. As a result, the research outcomes may be influenced to some extent by factors such as age and gender. Furthermore, there is a degree of variability in the uniformity of sample locations and grouping information across datasets. For instance, GSE168375 predominantly focuses on the temporal lobe, with grouping into epilepsy and control categories. In contrast, GSE186334 has a more detailed categorization (cortex_cytoplasm_ctrl, cortex_nucleus_ctrl, cortex_cytoplasm_mTLE, cortex_nucleus_mTLE). Given that our analysis is based on the grouping of epilepsy and control, variations in data conditions may also impact the results.

## Conclusion

In conclusion, our study provides valuable insights into TLE pathogenesis and proposes potential pharmacological interventions based on differentially expressed apoptotic genes. The constructed diagnostic model demonstrates high accuracy, laying the foundation for personalized treatment strategies. Despite limitations in sample size obtained through surgical procedures, future experimental validation is planned to enhance the robustness of our findings. Overall, this research contributes to advancing diagnostic and therapeutic approaches for epilepsy.

## Data availability statement

The datasets presented in this study can be found in online repositories. The names of the repository/repositories and accession number(s) can be found in this article/Supplementary material.

## Ethics statement

Ethical approval was not required for the study involving humans in accordance with the local legislation and institutional requirements. Written informed consent to participate in this study was not required from the participants or the participants’ legal guardians/next of kin in accordance with the national legislation and the institutional requirements.

## Author contributions

WW: Conceptualization, Data curation, Software, Writing – original draft. YR: Visualization, Writing – original draft. FX: Data curation, Software, Visualization, Writing – review & editing. XZ: Supervision, Validation, Writing – review & editing. FW: Supervision, Validation, Writing – review & editing. TW: Supervision, Validation, Writing – review & editing. HZ: Supervision, Validation, Writing – review & editing. XW: Conceptualization, Methodology, Project administration, Supervision, Writing – review & editing. YY: Conceptualization, Methodology, Project administration, Supervision, Writing – review & editing.

## Supplementary material

Supplementary Figure 1 KEGG pathways in the epilepsy group and the control group. (**A)** TNF signaling pathway. **(B)** Antigen processing and presentation pathway.

Supplementary Figure 2 GSVA results of epilepsy group and the control group. **(A)** Functional enrichment analysis in epilepsy group. **(B)** Spearman correlation between TGF_BETA_SIGNALING GSVA score and S100A9 expression. **(C)** Spearman correlation between REACTIVE_OXYGEN_SPECIES_PATHWAY GSVA score and S100A9 expression. **(D)** Spearman correlation between EPITHELIAL_MESENCHYMAL_TRANSITION GSVA score and S100A9 expression. “R” represents spearman correlation coefficient.

Supplementary Figure 3 GSVA analysis results for high and low-risk diagnostic groups. **(A)** Functional enrichment analysis in high and low-risk diagnostic groups. **(B)** Spearman correlation between GSVA score of GLYCOLYSIS pathway and risk diagnostic score. **(C)** Spearman correlation between GSVA score of MYC_TARGETS_V2 pathway and risk diagnostic score. **(D)** Spearman correlation between GSVA score of REACTIVE_OXYGEN_SPECIES_PATHWAY and risk diagnostic score. **(E)** Spearman correlation between GSVA score of TGF_BETA_SIGNALING pathway and risk diagnostic score. **(F)** Spearman correlation between GSVA score of EPITHELIAL_MESENCHYMAL_TRANSITION pathway and risk diagnostic score “R” represents spearman correlation coefficient.

Supplementary Figure 4 Diagnostic gene and miRNA and TFs network. **(A)** Network of diagnostic genes and miRNAs. **(B)** Network of diagnostic genes and TFs. TF, transcription factor.

Supplementary Figure 5 Spatial structure of the 5R8Q configuration of IL1B.
