# Supplementary material for: Identification of hub genes significantly linked to temporal lobe epilepsy and apoptosis via bioinformatics analysis
Source: Front Mol Neurosci. 2024 Feb 7;17:1300348. doi: 10.3389/fnmol.2024.1300348 (PMC10879302; doi:10.3389/fnmol.2024.1300348)
Supplement: Supplementary file 1 [file Table_1.docx]

# Supplementary table

## Table S1 Information of Datasets

| **Dataset** | **GSE168375** | **GSE186334** | **GSE140393** |
| --- | --- | --- | --- |
| **reference** | PMID34987109 | PMID35310884 | PMID36274170  PMID36443832 |
| **Organism** | Homo sapiens | Homo sapiens | Homo sapiens |
| **Experiment type** | Expression profiling by high throughput sequencing | Expression profiling by high throughput sequencing | Expression profiling by high throughput sequencing |
| **Platform** | GPL20301 Illumina HiSeq 4000 | GPL20301 Illumina HiSeq 4000 | GPL16791 Illumina HiSeq 2500 |
| **Tissue** | Brain (Cerebral cortex) | Brain (Cortex) | Temporal neocortex |
| **No. Samples associated with epilepsy** | Total: 43 (31 epilepsy samples vs 12 normal/healthy control samples) | Total: 36 ( 24 epilepsy samples vs 12 healthy control samples) | Total: 21 (12 epilepsy samples vs 9 healthy control samples) |

## Table S2 Apoptosis-related genes

| **Gene** | **Gene** | **Gene** | **Gene** | **Gene** | **Gene** |
| --- | --- | --- | --- | --- | --- |
| AATF | DDIT4 | KDM1A | PTGIS | TRIM32 | PIAS4 |
| ABL1 | DDX3X | KITLG | PTH | TRIM39 | PIDD1 |
| ACAA2 | DDX47 | KRT18 | PTPMT1 | TXNDC12 | PIH1D1 |
| ACKR3 | DDX5 | KRT8 | PTPN1 | TYROBP | PIK3R1 |
| ACVR1 | DEDD | LCK | PTPN2 | UACA | PINK1 |
| ACVR1B | DEDD2 | LGALS12 | PTPRC | UBB | PLAGL2 |
| ADORA1 | DELE1 | LGALS3 | PTTG1IP | UBE2K | PLAUR |
| AEN | DEPTOR | LRRK2 | PYCARD | UBE4B | PLEKHF1 |
| AGT | DIABLO | LTBR | QARS1 | UBQLN1 | PLSCR3 |
| AGTR2 | DIDO1 | LY96 | RACK1 | UMOD | PMAIP1 |
| AIFM1 | DNAJA1 | MADD | RAF1 | UNC5B | PML |
| AKT1 | DNAJC10 | MAEL | RB1 | URI1 | POLB |
| ANXA6 | DNM1L | MAGEA3 | RB1CC1 | USP28 | POU4F1 |
| APAF1 | DPF2 | MAP2K5 | RBCK1 | USP47 | POU4F2 |
| APPL1 | DYRK2 | MAP3K5 | RELA | VDAC2 | PPARD |
| AR | E2F1 | MAPK7 | RET | VNN1 | PPIA |
| ARHGEF2 | E2F2 | MAPK8 | RFFL | WDR35 | PPIF |
| ARL6IP5 | EDA2R | MAPK8IP1 | RHOT1 | WNT4 | PPM1F |
| ARMC10 | EIF2AK3 | MAPK8IP2 | RHOT2 | WWOX | PPP1CA |
| ARRB2 | ELL3 | MAPK9 | RIPK1 | XBP1 | PPP1R13B |
| ASAH2 | ENO1 | MARCHF7 | RIPK3 | YAP1 | PPP1R15A |
| ATF3 | EP300 | MAZ | RNF183 | YBX3 | PPP2R1B |
| ATF4 | EPHA2 | MCL1 | RNF186 | YWHAB | PPP3CC |
| ATM | EPO | MDM2 | RNF34 | YWHAE | PPP3R1 |
| ATP2A1 | ERBB3 | MELK | RNF41 | YWHAG | PRDX2 |
| ATP2A3 | ERCC6 | MFF | RPL11 | YWHAH | PRELID1 |
| ATP5IF1 | ERN1 | MIF | RPL26 | YWHAQ | PRKCA |
| AVP | ERN2 | MIR132 | RPS27L | YWHAZ | PRKCD |
| BAD | ERO1A | MIR15A | RPS3 | ZC3HC1 | PRKDC |
| BAG3 | ERP29 | MIR16-1 | RPS6KB1 | ZDHHC3 | PRKN |
| BAG5 | EYA1 | MIR17 | RPS7 | ZMYND11 | PRKRA |
| BAG6 | EYA2 | MIR198 | RRP8 | ZNF205 | PRODH |
| BAK1 | EYA3 | MIR21 | RTKN2 | ZNF385A | PSEN1 |
| BAX | EYA4 | MIR210 | RTL10 | ZNF385B | PSMD10 |
| BBC3 | FADD | MIR221 | S100A8 | ZNF622 | PSME3 |
| BCAP31 | FAF1 | MIR222 | S100A9 | ZSWIM2 | PTEN |
| BCL10 | FAIM | MIR26B | SCG2 | TLR3 | COA8 |
| BCL2 | FAIM2 | MIR27B | SCN2A | TLR4 | COL2A1 |
| BCL2A1 | FAM162A | MIR449A | SCRT2 | TM2D1 | CRADD |
| BCL2L1 | FAS | MKNK2 | SELENOK | TMBIM1 | CREB3 |
| BCL2L10 | FASLG | MLH1 | SELENOS | TMBIM6 | CREB3L1 |
| BCL2L11 | FASTK | MLLT11 | SENP1 | TMC8 | CRH |
| BCL2L12 | FBH1 | MMP9 | SEPTIN4 | TMEM102 | CRIP1 |
| BCL2L14 | FBXW7 | MNT | SERINC3 | TMEM109 | CSF2 |
| BCL2L2 | FEM1B | MOAP1 | SERPINE1 | TMEM117 | CSNK2A1 |
| BCL3 | FGA | MPV17L | SFN | TMEM14A | CSNK2A2 |
| BCLAF1 | FGB | MSH2 | SFPQ | TMEM161A | CTH |
| BDKRB2 | FGF10 | MSH6 | SFRP1 | TNF | CTNNA1 |
| BDNF | FGFR1 | MSX1 | SFRP2 | TNFAIP3 | CTSC |
| BECN1 | FGFR3 | MUC1 | SGMS1 | TNFRSF10A | CTTN |
| BID | FGG | MUL1 | SGPL1 | TNFRSF10B | CUL1 |
| BIK | FHIT | MYBBP1A | SGPP1 | TNFRSF10C | CUL2 |
| BIRC6 | FIGNL1 | NACC2 | SH3RF1 | TNFRSF12A | CUL3 |
| BLOC1S2 | FIS1 | NANOS3 | SHH | TNFRSF1A | CUL4A |
| BMF | FNIP2 | NBN | SHISA5 | TNFRSF1B | CUL5 |
| BMP4 | FXN | NCK1 | SIAH1 | TNFRSF25 | CX3CL1 |
| BMP5 | FYN | NCK2 | SIAH2 | TNFSF10 | CX3CR1 |
| BMPR1B | FZD9 | NDUFA13 | SIRT1 | TNFSF12 | CXCL12 |
| BNIP3 | G0S2 | NDUFS3 | SIVA1 | TOPORS | CYLD |
| BNIP3L | GABARAP | NFATC4 | SKIL | TP53 | CYP1B1 |
| BOK | GATA1 | NFE2L2 | SLC25A5 | TP53BP2 | DAB2IP |
| BRCA1 | GATA4 | NGF | SLC35F6 | TP63 | DAP |
| BRCA2 | GCLM | NGFR | SLC9A3R1 | TP73 | DAP3 |
| BRSK2 | GDNF | NKX3-1 | SMAD3 | TPD52L1 | DAPK1 |
| BTK | GFRAL | NLE1 | SNAI1 | TPT1 | DAPK2 |
| CAAP1 | GGCT | NME5 | SNAI2 | TRADD | DAPK3 |
| CASP1 | GHITM | NMT1 | SNW1 | TRAF1 | DAPL1 |
| CASP10 | GNAI2 | NOC2L | SOD1 | TRAF2 | DAXX |
| CASP12 | GNAI3 | NOG | SOD2 | TRAF7 | DBH |
| CASP2 | GPER1 | NOL3 | SORT1 | TRAP1 | DCC |
| CASP3 | GPX1 | NONO | SP100 | TRIAP1 | DDIAS |
| CASP4 | GRINA | NOS3 | SRC | TRIB3 | DDIT3 |
| CASP5 | GSDME | NOX1 | SRPX | ICAM1 | ITGAV |
| CASP8 | GSK3A | NR4A2 | SST | IFI16 | ITM2C |
| CASP8AP2 | GSK3B | NUPR1 | SSTR3 | IFI27 | ITPR1 |
| CASP9 | GSKIP | OPA1 | ST20 | IFI27L1 | ITPRIP |
| CAV1 | GSTP1 | P2RX4 | STK11 | IFI27L2 | IVNS1ABP |
| CCAR2 | GZMB | P2RX7 | STK24 | IFI6 | JAK2 |
| CCK | HDAC1 | P4HB | STK25 | IFNB1 | JMY |
| CD14 | HERPUD1 | PAK2 | STK3 | IFNG | JUN |
| CD24 | HGF | PAK5 | STK4 | IGF1 |  |
| CD27 | HIC1 | PARK7 | STRADB | IKBKE |  |
| CD28 | HIF1A | PARP1 | STX4 | IL12A |  |
| CD38 | HINT1 | PARP2 | STYXL1 | IL19 |  |
| CD3E | HIP1 | PAWR | SYVN1 | IL1A |  |
| CD44 | HIP1R | PCGF2 | TAF9 | IL1B |  |
| CD5 | HIPK1 | PDCD10 | TAF9B | IL2 |  |
| CD70 | HIPK2 | PDCD5 | TCF7L2 | IL20RA |  |
| CD74 | HMGB2 | PDCD6 | TERT | IL33 |  |
| CDIP1 | HMOX1 | PDIA3 | TFDP1 | IL4 |  |
| CDKN1A | HNRNPK | PDK1 | TFDP2 | IL6R |  |
| CDKN2A | HRAS | PDK2 | TFPT | IL7 |  |
| CEBPB | HRK | PDPK1 | TGFB1 | INCA1 |  |
| CFLAR | HSPA1A | PDX1 | TGFB2 | ING2 |  |
| CHAC1 | HSPA1B | PEA15 | TGFBR1 | ING5 |  |
| CHCHD10 | HSPB1 | PELI3 | THBS1 | INHBA |  |
| CHEK2 | HTRA2 | PERP | TICAM1 | INHBB |  |
| CIB1 | HTT | PF4 | TICAM2 | INS |  |
| CIDEB | HYAL2 | PHIP | TIMM50 | ITGA6 |  |
| CLU | HYOU1 | PHLDA3 | TIMP3 | ITGAM |  |

## Table S3 GO enrichment results based on differentially expressed apoptosis-related genes of epilepsy group and control group in combined dataset

| **Ontology** | **Description** | **P adjust** |
| --- | --- | --- |
| BP | regulation of apoptotic signaling pathway | 7.81E-15 |
| BP | negative regulation of apoptotic signaling pathway | 4.55E-11 |
| BP | positive regulation of NF-kappaB transcription factor activity | 1.00E-10 |
| BP | negative regulation of extrinsic apoptotic signaling pathway | 3.66E-10 |
| BP | positive regulation of DNA-binding transcription factor activity | 4.93E-09 |
| BP | regulation of extrinsic apoptotic signaling pathway | 5.94E-09 |
| BP | leukocyte aggregation | 5.31E-08 |
| BP | extrinsic apoptotic signaling pathway | 5.95E-08 |
| BP | regulation of DNA-binding transcription factor activity | 1.92E-07 |
| BP | positive regulation of interleukin-8 production | 2.26E-07 |
| BP | negative regulation of signal  transduction in absence of ligand | 2.07E-06 |
| BP | negative regulation of extrinsic  apoptotic signaling pathway in absence of ligand | 2.07E-06 |
| BP | regulation of interleukin-8 production | 2.07E-06 |
| BP | interleukin-8 production | 2.07E-06 |
| BP | leukocyte cell-cell adhesion | 2.25E-06 |
| BP | astrocyte development | 3.58E-06 |
| BP | positive regulation of response to external stimulus | 4.35E-06 |
| BP | regulation of extrinsic apoptotic  signaling pathway in absence of ligand | 4.63E-06 |
| BP | glial cell activation | 8.47E-06 |
| BP | positive regulation of defense response | 9.31E-06 |
| BP | positive regulation of inflammatory response | 9.92E-06 |
| BP | response to lipopolysaccharide | 1.49E-05 |
| BP | positive regulation of heterotypic cell-cell adhesion | 1.49E-05 |
| …… | …… | …… |
| CC | aggresome | 1.44E-02 |
| CC | inclusion body | 1.44E-02 |
| CC | secretory granule membrane | 1.44E-02 |
| CC | secretory granule lumen | 1.44E-02 |
| CC | cytoplasmic vesicle lumen | 1.44E-02 |
| CC | vesicle lumen | 1.44E-02 |
| CC | nuclear speck | 1.74E-02 |
| CC | focal adhesion | 1.74E-02 |
| CC | ficolin-1-rich granule lumen | 1.74E-02 |
| CC | cell-substrate junction | 1.74E-02 |
| CC | collagen-containing extracellular matrix | 1.74E-02 |
| CC | blood microparticle | 2.03E-02 |
| CC | neuronal cell body | 2.03E-02 |
| CC | centriole | 2.03E-02 |
| CC | peptidase inhibitor complex | 2.41E-02 |
| CC | ficolin-1-rich granule | 2.43E-02 |
| CC | basolateral plasma membrane | 3.44E-02 |
| CC | basal plasma membrane | 3.79E-02 |
| CC | lamellipodium membrane | 3.79E-02 |
| CC | basal part of cell | 4.05E-02 |
| CC | phagocytic cup | 4.31E-02 |
| CC | neuronal cell body membrane | 4.31E-02 |
| CC | cell body membrane | 4.54E-02 |
| CC | membrane raft | 4.56E-02 |
| CC | membrane microdomain | 4.56E-02 |
| …… | …… | …… |
| MF | RAGE receptor binding | 1.38E-03 |
| MF | Toll-like receptor binding | 1.38E-03 |
| MF | protein-containing complex destabilizing activity | 1.38E-03 |
| MF | long-chain fatty acid binding | 1.64E-03 |
| MF | misfolded protein binding | 5.04E-03 |
| MF | ATP-dependent protein folding chaperone | 8.42E-03 |
| MF | fatty acid binding | 1.03E-02 |
| MF | protein folding chaperone | 1.40E-02 |
| MF | cytokine receptor binding | 1.40E-02 |
| MF | G protein-coupled receptor binding | 1.40E-02 |
| MF | virus receptor activity | 1.40E-02 |
| MF | exogenous protein binding | 1.40E-02 |
| MF | monocarboxylic acid binding | 1.40E-02 |
| MF | calcium-dependent protein binding | 1.40E-02 |
| MF | cytokine receptor activity | 1.86E-02 |
| MF | protein N-terminus binding | 2.07E-02 |
| MF | heat shock protein binding | 2.50E-02 |
| MF | unfolded protein binding | 2.50E-02 |
| MF | histone deacetylase binding | 2.52E-02 |
| MF | organic acid binding | 2.68E-02 |

GO: Gene ontology; MF: Molecular Function; BP: Biological Process; CC: Cell Component.

## Table S4 KEGG enrichment results based on differentially expressed apoptosis-related genes of epilepsy group and control group in combined dataset

| **ID** | **Description** | **P adjust** |
| --- | --- | --- |
| hsa05134 | Legionellosis | 1.12E-04 |
| hsa04657 | IL-17 signaling pathway | 3.68E-04 |
| hsa04640 | Hematopoietic cell lineage | 3.68E-04 |
| hsa04612 | Antigen processing and presentation | 4.60E-03 |
| hsa05417 | Lipid and atherosclerosis | 4.60E-03 |
| hsa04933 | AGE-RAGE signaling pathway in diabetic complications | 6.12E-03 |
| hsa05142 | Chagas disease | 6.12E-03 |
| hsa05145 | Toxoplasmosis | 6.27E-03 |
| hsa05020 | Prion disease | 6.27E-03 |
| hsa04010 | MAPK signaling pathway | 7.51E-03 |
| hsa04380 | Osteoclast differentiation | 7.51E-03 |
| hsa01523 | Antifolate resistance | 7.51E-03 |
| hsa05162 | Measles | 7.97E-03 |
| hsa05143 | African trypanosomiasis | 9.79E-03 |
| hsa05332 | Graft-versus-host disease | 1.16E-02 |
| hsa04940 | Type I diabetes mellitus | 1.16E-02 |
| hsa05144 | Malaria | 1.47E-02 |
| hsa04213 | Longevity regulating pathway - multiple species | 2.05E-02 |
| hsa05321 | Inflammatory bowel disease | 2.20E-02 |
| hsa05131 | Shigellosis | 2.65E-02 |
| hsa05133 | Pertussis | 2.65E-02 |
| hsa05140 | Leishmaniasis | 2.65E-02 |
| hsa05323 | Rheumatoid arthritis | 3.59E-02 |
| hsa04060 | Cytokine-cytokine receptor interaction | 3.59E-02 |
| hsa04061 | Viral protein interaction with cytokine and cytokine receptor | 3.59E-02 |
| hsa05146 | Amoebiasis | 3.59E-02 |
| hsa04064 | NF-kappa B signaling pathway | 3.59E-02 |
| hsa04620 | Toll-like receptor signaling pathway | 3.59E-02 |
| hsa04625 | C-type lectin receptor signaling pathway | 3.59E-02 |
| hsa04668 | TNF signaling pathway | 4.14E-02 |

KEGG: Kyoto Encyclopedia of Genes and Genomes

## Table S5 GSEA enrichment results based on differentially expressed apoptosis-related genes of epilepsy group and control group in combined dataset

| **Description** | **NES** | **P adjust** |
| --- | --- | --- |
| KEGG_TGF_BETA_SIGNALING_PATHWAY | 1.966449 | 5.36E-04 |
| KEGG_CYTOKINE_CYTOKINE_RECEPTOR_INTERACTION | 1.723296 | 5.36E-04 |
| KEGG_COMPLEMENT_AND_COAGULATION_CASCADES | 1.865589 | 4.64E-03 |
| KEGG_ECM_RECEPTOR_INTERACTION | 1.715724 | 1.65E-02 |
| KEGG_GRAFT_VERSUS_HOST_DISEASE | 1.77505 | 2.19E-02 |
| KEGG_TYPE_I_DIABETES_MELLITUS | 1.810511 | 2.23E-02 |
| KEGG_HEMATOPOIETIC_CELL_LINEAGE | 1.660849 | 2.23E-02 |
| KEGG_PATHWAYS_IN_CANCER | 1.453662 | 2.23E-02 |
| KEGG_RIBOSOME | 1.661579 | 2.73E-02 |

GSEA: Gene Set Enrichment Analysis

## Table S6 Differential pathways of epilepsy group and control group based on GSVA analysis

| **ID** | **T** | **P value** |
| --- | --- | --- |
| HALLMARK_HEME_METABOLISM | -2.4085 | 1.82E-02 |
| HALLMARK_REACTIVE_  OXYGEN_SPECIES_PATHWAY | -2.39868 | 1.86E-02 |
| HALLMARK_KRAS_SIGNALING_UP | 2.328036 | 2.23E-02 |
| HALLMARK_KRAS_SIGNALING_DN | -2.31292 | 2.31E-02 |
| HALLMARK_TGF_BETA_SIGNALING | 2.252332 | 2.69E-02 |
| HALLMARK_EPITHELIAL_  MESENCHYMAL_TRANSITION | 1.988612 | 5.00E-02 |
| HALLMARK_P53_PATHWAY | -1.85623 | 6.69E-02 |
| HALLMARK_COAGULATION | 1.802147 | 7.51E-02 |
| HALLMARK_MYC_TARGETS_V2 | -1.69386 | 9.39E-02 |
| HALLMARK_CHOLESTEROL_HOMEOSTASIS | 1.584202 | 1.17E-01 |
| HALLMARK_GLYCOLYSIS | -1.51101 | 1.34E-01 |
| HALLMARK_UV_RESPONSE_DN | 1.469765 | 1.45E-01 |
| HALLMARK_HEDGEHOG_SIGNALING | -1.40038 | 1.65E-01 |
| HALLMARK_COMPLEMENT | 1.318948 | 1.91E-01 |
| HALLMARK_PEROXISOME | -1.21889 | 2.26E-01 |
| HALLMARK_MYOGENESIS | -1.17921 | 2.42E-01 |
| HALLMARK_ANDROGEN_RESPONSE | 1.123462 | 2.64E-01 |
| HALLMARK_INFLAMMATORY_RESPONSE | 1.102445 | 2.73E-01 |
| HALLMARK_MTORC1_SIGNALING | 1.087706 | 2.80E-01 |
| HALLMARK_ALLOGRAFT_REJECTION | 1.087068 | 2.80E-01 |
| HALLMARK_MITOTIC_SPINDLE | -1.03334 | 3.04E-01 |
| HALLMARK_APOPTOSIS | 0.968321 | 3.36E-01 |
| HALLMARK_NOTCH_SIGNALING | 0.962035 | 3.39E-01 |
| HALLMARK_TNFA_SIGNALING_VIA_NFKB | 0.959051 | 3.40E-01 |
| HALLMARK_INTERFERON_GAMMA_RESPONSE | 0.890161 | 3.76E-01 |
| HALLMARK_PROTEIN_SECRETION | 0.843415 | 4.01E-01 |
| HALLMARK_MYC_TARGETS_V1 | 0.785782 | 4.34E-01 |
| HALLMARK_ESTROGEN_RESPONSE_EARLY | -0.77655 | 4.40E-01 |
| HALLMARK_INTERFERON_ALPHA_RESPONSE | 0.740257 | 4.61E-01 |
| HALLMARK_UV_RESPONSE_UP | -0.72851 | 4.68E-01 |
| HALLMARK_XENOBIOTIC_METABOLISM | -0.71923 | 4.74E-01 |
| HALLMARK_IL6_JAK_STAT3_SIGNALING | 0.672476 | 5.03E-01 |
| HALLMARK_E2F_TARGETS | 0.650879 | 5.17E-01 |
| HALLMARK_HYPOXIA | -0.63339 | 5.28E-01 |
| HALLMARK_ANGIOGENESIS | 0.591662 | 5.56E-01 |
| HALLMARK_DNA_REPAIR | -0.57003 | 5.70E-01 |
| HALLMARK_PANCREAS_BETA_CELLS | 0.565462 | 5.73E-01 |
| HALLMARK_ADIPOGENESIS | -0.5576 | 5.79E-01 |
| HALLMARK_FATTY_ACID_METABOLISM | 0.540866 | 5.90E-01 |
| HALLMARK_SPERMATOGENESIS | -0.50714 | 6.13E-01 |
| HALLMARK_APICAL_SURFACE | 0.379023 | 7.06E-01 |
| HALLMARK_APICAL_JUNCTION | -0.37096 | 7.12E-01 |
| HALLMARK_G2M_CHECKPOINT | 0.319059 | 7.50E-01 |
| HALLMARK_OXIDATIVE_PHOSPHORYLATION | -0.26474 | 7.92E-01 |
| HALLMARK_UNFOLDED_PROTEIN_RESPONSE | 0.237548 | 8.13E-01 |
| HALLMARK_BILE_ACID_METABOLISM | -0.18635 | 8.53E-01 |
| HALLMARK_PI3K_AKT_MTOR_SIGNALING | 0.088263 | 9.30E-01 |
| HALLMARK_WNT_BETA_CATENIN_SIGNALING | -0.07537 | 9.40E-01 |
| HALLMARK_IL2_STAT5_SIGNALING | -0.07274 | 9.42E-01 |
| HALLMARK_ESTROGEN_RESPONSE_LATE | -0.01556 | 9.88E-01 |

GSVA: Gene Set Variation analysis

## Table S7 Gene-TF relationship

| **ID** | **Target** | **Experiment** | **Literature** |
| --- | --- | --- | --- |
| AHR | IL1B | Unknown | 23349129 |
| CEBPB | IL1B | + | 17386941 |
| CEBPB | IL1B | Unknown | 10383163;10801783 |
| E2F1 | IL1B | Unknown | 17707233 |
| HMGA1 | IL1B | Unknown | 15901130 |
| HSF1 | IL1B | - | 10328874 |
| IRF8 | IL1B | + | 17386941 |
| JUN | IL1B | Unknown | 11306276 |
| JUNB | IL1B | Unknown | 11306276 |
| KLF4 | IL1B | - | 22449968 |
| MYC | CD38 | + | 11519042 |
| NFIL3 | IL1B | - | 8547328 |
| NFKB1 | CD38 | Unknown | 17322278;18341691;18441094 |
| NFKB1 | IL1B | + | 12686724;14960579;8413223;8679226;9878621 |
| NFKB1 | IL1B | Unknown | 10080875;10963848;16433741;  17707233;18285351;20336759;9058643 |
| NFKBIA | IL1B | - | 15228586 |
| REL | IL1B | + | 8413223 |
| RELA | CD38 | Unknown | 17322278;18341691;18441094 |
| RELA | IL1B | + | 12686724;14960579;8021507;  8413223;8679226;9878621 |
| RELA | IL1B | Unknown | 10080875;10963848;16433741;  17707233;18285351;20336759;9058643 |
| SIRT1 | IL1B | Unknown | 21245135 |
| SPI1 | IL1B | + | 17386941 |
| SPI1 | IL1B | Unknown | 10801783 |
| STAT1 | IL1B | + | 17386941 |
| SUGP1 | IL1B | + | 9878621 |
| YY1 | IL1B | Unknown | 22467534 |

TF: Transcription Factor

## Table S8 Gene-miRNA relationship

| **ID** | **Target** | **Experiment** | **Literature** |
| --- | --- | --- | --- |
| hsa-mir-21-5p | IL1B | Luciferase reporter assay//  Microarray//  qRT-PCR | 21131358 |
| hsa-mir-24-3p | IL1B | Microarray//qRT-PCR | 28057018 |
| hsa-mir-24-3p | S100A8 | Luciferase reporter assay//  Western blot | 22139384 |
| hsa-mir-25-3p | PAWR | PAR-CLIP | 23446348\|  21572407\|  20371350 |
| hsa-mir-30a-5p | PAWR | HITS-CLIP | 22473208 |
| hsa-mir-32-5p | PAWR | PAR-CLIP | 23446348\|  21572407\|  20371350 |
| hsa-mir-92a-3p | PAWR | PAR-CLIP | 23446348\|  21572407\|  20371350 |
| hsa-mir-98-5p | S100A8 | Microarray | 19088304 |
| hsa-mir-103a-3p | PAWR | PAR-CLIP | 23446348\|  21572407\|  20371350 |
| hsa-mir-106a-5p | IL1B | qRT-PCR | 25950430 |
| hsa-mir-107 | PAWR | PAR-CLIP | 23446348\|  21572407\|  20371350 |
| hsa-mir-192-5p | PAWR | Microarray | 19074876 |
| hsa-mir-30c-5p | PAWR | HITS-CLIP | 22473208 |
| hsa-mir-30d-5p | PAWR | HITS-CLIP | 22473208 |
| hsa-mir-204-5p | IL1B | Microarray | 21282569 |
| hsa-mir-215-5p | PAWR | Microarray | 19074876 |
| hsa-mir-218-5p | PAWR | PAR-CLIP | 23446348\|  21572407\|  20371350 |
| hsa-mir-30b-5p | PAWR | HITS-CLIP | 22473208 |
| hsa-mir-125b-5p | S100A8 | CLASH | 23622248 |
| hsa-mir-30e-5p | PAWR | HITS-CLIP | 22473208 |
| hsa-mir-363-3p | PAWR | PAR-CLIP | 23446348\|  21572407\|  20371350 |
| hsa-mir-367-3p | PAWR | PAR-CLIP | 23446348\|  21572407\|  20371350 |
| hsa-mir-335-5p | CD38 | Microarray | 18185580 |
| hsa-mir-488-5p | PAWR | PAR-CLIP | 20371350 |
| hsa-mir-496 | PAWR | PAR-CLIP | 20371350 |
| hsa-mir-493-3p | PAWR | PAR-CLIP | 20371350 |
| hsa-mir-92b-3p | PAWR | PAR-CLIP | 23446348\|  21572407\|  20371350 |
| hsa-mir-591 | PAWR | PAR-CLIP | 21572407 |
| hsa-mir-621 | PAWR | PAR-CLIP | 23446348\|  21572407\|  20371350 |
| hsa-mir-632 | FAIM2 | HITS-CLIP | 23824327 |
| hsa-mir-653-5p | PAWR | PAR-CLIP | 20371350 |
| hsa-mir-659-3p | CD38 | PAR-CLIP | 23446348\|  21572407 |
| hsa-mir-19a-5p | CD38 | PAR-CLIP | 23446348\|  21572407 |
| hsa-mir-19b-1-5p | CD38 | PAR-CLIP | 23446348\|  21572407 |
| hsa-mir-19b-2-5p | CD38 | PAR-CLIP | 23446348\|  21572407 |
| hsa-mir-101-5p | PAWR | PAR-CLIP | 21572407 |
| hsa-mir-218-1-3p | CD38 | PAR-CLIP | 23446348\|  21572407 |
| hsa-mir-124-5p | PAWR | PAR-CLIP | 20371350 |
| hsa-mir-140-3p | CD38 | Luciferase reporter assay//  qRT-PCR | 22773691 |
| hsa-mir-186-3p | PAWR | PAR-CLIP | 20371350 |
| hsa-mir-590-3p | PAWR | PAR-CLIP | 20371350 |
| hsa-mir-875-3p | FAIM2 | HITS-CLIP | 23824327 |
| hsa-mir-877-3p | IL1B | Luciferase reporter assay | 27542871 |
| hsa-mir-887-3p | IL1B | Luciferase reporter assay | 27542871 |
| hsa-mir-1231 | FAIM2 | HITS-CLIP | 23824327 |
| hsa-mir-1301-3p | PAWR | PAR-CLIP | 23446348\|  21572407\|  20371350 |
| hsa-mir-1179 | PAWR | PAR-CLIP | 21572407\|  20371350 |
| hsa-mir-1299 | FAIM2 | HITS-CLIP | 23824327 |
| hsa-mir-548l | PAWR | PAR-CLIP | 20371350 |
| hsa-mir-548n | PAWR | PAR-CLIP | 20371350 |
| hsa-mir-1267 | PAWR | PAR-CLIP | 20371350 |
| hsa-mir-4307 | PAWR | PAR-CLIP | 20371350 |
| hsa-mir-4267 | FAIM2 | HITS-CLIP | 23824327 |
| hsa-mir-4282 | PAWR | PAR-CLIP | 20371350 |
| hsa-mir-4288 | FAIM2 | HITS-CLIP | 23824327 |
| hsa-mir-3607-5p | PAWR | PAR-CLIP | 20371350 |
| hsa-mir-3611 | PAWR | PAR-CLIP | 20371350 |
| hsa-mir-3613-3p | CD38 | PAR-CLIP | 23446348\|  21572407 |
| hsa-mir-3646 | PAWR | PAR-CLIP | 20371350 |
| hsa-mir-3655 | FAIM2 | HITS-CLIP | 23824327 |
| hsa-mir-3692-3p | PAWR | PAR-CLIP | 20371350 |
| hsa-mir-548ag | PAWR | PAR-CLIP | 20371350 |
| hsa-mir-4461 | PAWR | PAR-CLIP | 20371350 |
| hsa-mir-548ai | PAWR | PAR-CLIP | 20371350 |
| hsa-mir-3152-5p | FAIM2 | HITS-CLIP | 23824327 |
| hsa-mir-4659a-5p | CD38 | PAR-CLIP | 23446348\|  21572407 |
| hsa-mir-4659b-5p | CD38 | PAR-CLIP | 23446348\|  21572407 |
| hsa-mir-4680-5p | PAWR | PAR-CLIP | 20371350 |
| hsa-mir-203b-3p | PAWR | PAR-CLIP | 20371350 |
| hsa-mir-4733-5p | PAWR | PAR-CLIP | 20371350 |
| hsa-mir-4776-3p | CD38 | PAR-CLIP | 23446348\|  21572407 |
| hsa-mir-5047 | PAWR | PAR-CLIP | 23446348\|  21572407\|  20371350 |
| hsa-mir-1273g-5p | CD38 | PAR-CLIP | 23446348\|  21572407 |
| hsa-mir-5586-5p | PAWR | PAR-CLIP | 20371350 |
| hsa-mir-5590-5p | CD38 | PAR-CLIP | 23446348\|  21572407 |
| hsa-mir-5692a | CD38 | PAR-CLIP | 23446348\|  21572407 |
| hsa-mir-570-5p | PAWR | PAR-CLIP | 20371350 |
| hsa-mir-6128 | FAIM2 | HITS-CLIP | 23824327 |
| hsa-mir-6512-5p | PAWR | PAR-CLIP | 21572407 |
| hsa-mir-95-5p | PAWR | PAR-CLIP | 20371350 |
| hsa-mir-1252-3p | PAWR | PAR-CLIP | 20371350 |
| hsa-mir-6778-3p | FAIM2 | HITS-CLIP | 23824327 |
| hsa-mir-6791-3p | FAIM2 | HITS-CLIP | 23824327 |
| hsa-mir-6829-3p | FAIM2 | HITS-CLIP | 23824327 |
| hsa-mir-6836-3p | FAIM2 | HITS-CLIP | 23824327 |
| hsa-mir-6869-5p | PAWR | PAR-CLIP | 20371350 |
| hsa-mir-7156-3p | PAWR | PAR-CLIP | 23446348\|  21572407\|  20371350 |
| hsa-mir-7158-3p | PAWR | PAR-CLIP | 20371350 |
| hsa-mir-8066 | PAWR | PAR-CLIP | 20371350 |
| hsa-mir-8084 | PAWR | PAR-CLIP | 20371350 |
| hsa-mir-548ba | PAWR | PAR-CLIP | 20371350 |
| hsa-mir-7977 | FAIM2 | HITS-CLIP | 23824327 |
| hsa-mir-203a-5p | CD38 | PAR-CLIP | 23446348\|  21572407 |
| hsa-mir-101-3p | CD38 | PAR-CLIP | tarbase |
| hsa-mir-106b-5p | CD38 | PAR-CLIP | tarbase |
| hsa-mir-17-5p | CD38 | PAR-CLIP | tarbase |
| hsa-mir-20a-5p | CD38 | PAR-CLIP | tarbase |
| hsa-mir-21-5p | CD38 | PAR-CLIP | tarbase |
| hsa-mir-3940-5p | CD38 | PAR-CLIP | tarbase |
| hsa-mir-92a-1-5p | CD38 | PAR-CLIP | tarbase |
| hsa-mir-1343-3p | CD38 | RNA-Seq | tarbase |
| hsa-mir-129-2-3p | CD38 | Microarrays | tarbase |
| hsa-mir-26a-5p | CD38 | Microarrays | tarbase |
| hsa-mir-27a-3p | CD38 | Microarrays | tarbase |
| hsa-mir-155-5p | IL1B | ELISA | tarbase |
| hsa-mir-181c-3p | IL1B | HITS-CLIP | tarbase |
| hsa-mir-376c-3p | IL1B | Microarrays, qPCR | tarbase |
| hsa-mir-587 | IL1B | HITS-CLIP | tarbase |
| hsa-mir-101-3p | IL1B | Microarrays | tarbase |
| hsa-mir-10b-5p | IL1B | Microarrays | tarbase |
| hsa-mir-126-3p | IL1B | Microarrays | tarbase |
| hsa-mir-128-3p | IL1B | Microarrays | tarbase |
| hsa-mir-129-2-3p | IL1B | Microarrays | tarbase |
| hsa-mir-203a-3p | IL1B | Microarrays | tarbase |
| hsa-mir-34a-5p | IL1B | Microarrays | tarbase |
| hsa-mir-34c-5p | IL1B | Microarrays | tarbase |
| hsa-mir-375 | IL1B | Microarrays | tarbase |
| hsa-mir-429 | IL1B | Microarrays | tarbase |
| hsa-mir-449a | IL1B | Microarrays | tarbase |
| hsa-mir-7-5p | IL1B | Microarrays | tarbase |
| hsa-mir-10b-5p | FAIM2 | Microarrays | tarbase |
| hsa-mir-16-5p | FAIM2 | Microarrays | tarbase |
| hsa-mir-320b | FAIM2 | Microarrays | tarbase |
| hsa-mir-346 | S100A8 | HITS-CLIP | tarbase |
| hsa-mir-1343-3p | S100A8 | RNA-Seq | tarbase |
| hsa-mir-126-3p | S100A8 | Microarrays | tarbase |
| hsa-mir-20a-5p | S100A8 | Microarrays | tarbase |
| hsa-mir-34a-5p | S100A8 | Microarrays | tarbase |
| hsa-mir-34b-5p | S100A8 | Microarrays | tarbase |
| hsa-mir-34c-5p | S100A8 | Microarrays | tarbase |
| hsa-mir-449b-5p | S100A8 | Microarrays | tarbase |
| hsa-mir-671-5p | S100A8 | Microarrays | tarbase |
| hsa-mir-140-3p | PAWR | HITS-CLIP | tarbase |
| hsa-mir-155-5p | PAWR | PAR-CLIP, Microarrays | tarbase |
| hsa-mir-181a-5p | PAWR | PAR-CLIP, HITS-CLIP | tarbase |
| hsa-mir-181b-5p | PAWR | HITS-CLIP | tarbase |
| hsa-mir-181c-5p | PAWR | PAR-CLIP | tarbase |
| hsa-mir-188-5p | PAWR | HITS-CLIP | tarbase |
| hsa-mir-193a-3p | PAWR | HITS-CLIP | tarbase |
| hsa-mir-193b-3p | PAWR | HITS-CLIP | tarbase |
| hsa-mir-200b-3p | PAWR | HITS-CLIP | tarbase |
| hsa-mir-30a-3p | PAWR | HITS-CLIP | tarbase |
| hsa-mir-30d-3p | PAWR | HITS-CLIP | tarbase |
| hsa-mir-30e-3p | PAWR | HITS-CLIP | tarbase |
| hsa-mir-3179 | PAWR | HITS-CLIP | tarbase |
| hsa-mir-339-3p | PAWR | HITS-CLIP | tarbase |
| hsa-mir-346 | PAWR | HITS-CLIP | tarbase |
| hsa-mir-362-5p | PAWR | HITS-CLIP | tarbase |
| hsa-mir-4705 | PAWR | HITS-CLIP | tarbase |
| hsa-mir-522-5p | PAWR | IMPACT-Seq | tarbase |
| hsa-mir-548b-3p | PAWR | HITS-CLIP | tarbase |
| hsa-mir-5585-3p | PAWR | PAR-CLIP | tarbase |
| hsa-mir-582-3p | PAWR | HITS-CLIP | tarbase |
| hsa-mir-625-3p | PAWR | HITS-CLIP | tarbase |
| hsa-mir-877-3p | PAWR | HITS-CLIP | tarbase |
| hsa-mir-93-5p | PAWR | PAR-CLIP | tarbase |
| hsa-mir-1-3p | PAWR | RPF-Seq, RNA-Seq | tarbase |
| hsa-mir-23b-3p | PAWR | RNA-Seq | tarbase |
| hsa-mir-124-3p | PAWR | Microarrays | tarbase |
| hsa-mir-126-3p | PAWR | Microarrays | tarbase |
| hsa-mir-129-2-3p | PAWR | Microarrays | tarbase |
| hsa-mir-191-5p | PAWR | Microarrays | tarbase |
| hsa-mir-210-3p | PAWR | Microarrays | tarbase |
| hsa-mir-376a-5p | PAWR | Microarrays | tarbase |

## Table S9 Gene-drug relationship

| **DrugBank.ID** | **Gene** | **Name** |
| --- | --- | --- |
| DB01017 | IL1B | Minocycline |
| DB01373 | S100A8 | Calcium |
| DB01593 | S100A8 | Zinc |
| DB05133 | IL1B | VP025 |
| DB05260 | IL1B | Gallium nitrate |
| DB05412 | IL1B | Talmapimod |
| DB05442 | IL1B | Etiprednol dicloacetate |
| DB05470 | IL1B | VX-702 |
| DB05507 | IL1B | VX-765 |
| DB05767 | IL1B | Andrographolide |
| DB09130 | S100A8 | Copper |
| DB11967 | IL1B | Binimetinib |
| DB12140 | IL1B | Dilmapimod |
| DB14487 | S100A8 | Zinc acetate |
| DB14533 | S100A8 | Zinc chloride |
