# Supplementary figures and images for: Identification of hub genes significantly linked to temporal lobe epilepsy and apoptosis via bioinformatics analysis
Source: Front Mol Neurosci. 2024 Feb 7;17:1300348. doi: 10.3389/fnmol.2024.1300348 (PMC10879302; doi:10.3389/fnmol.2024.1300348)

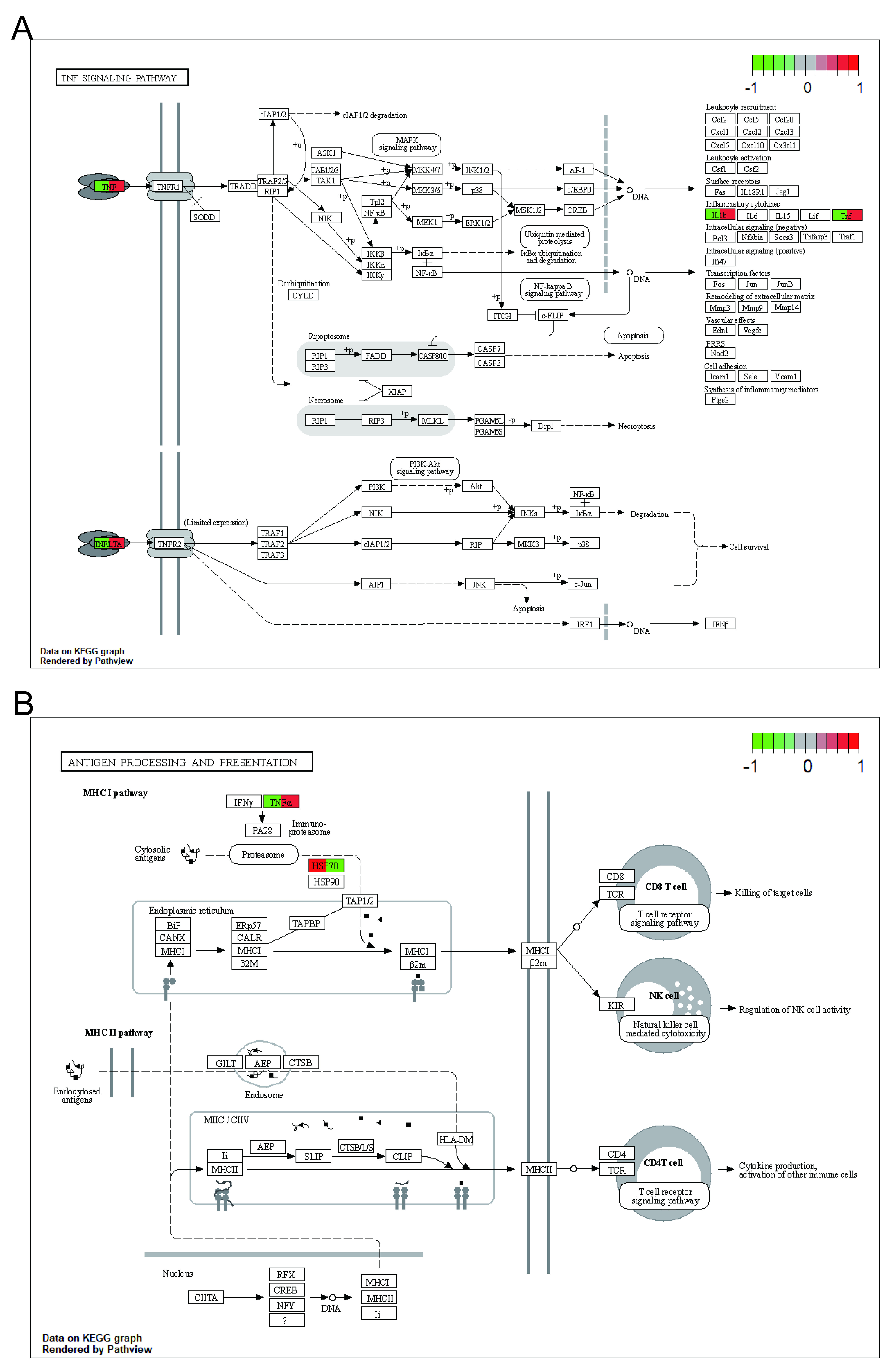

Supplement: Supplementary Figure 1 — KEGG pathways in the epilepsy group and the control group. (A) TNF signaling pathway. (B) Antigen processing and presentation pathway. [file Image_1.JPEG]

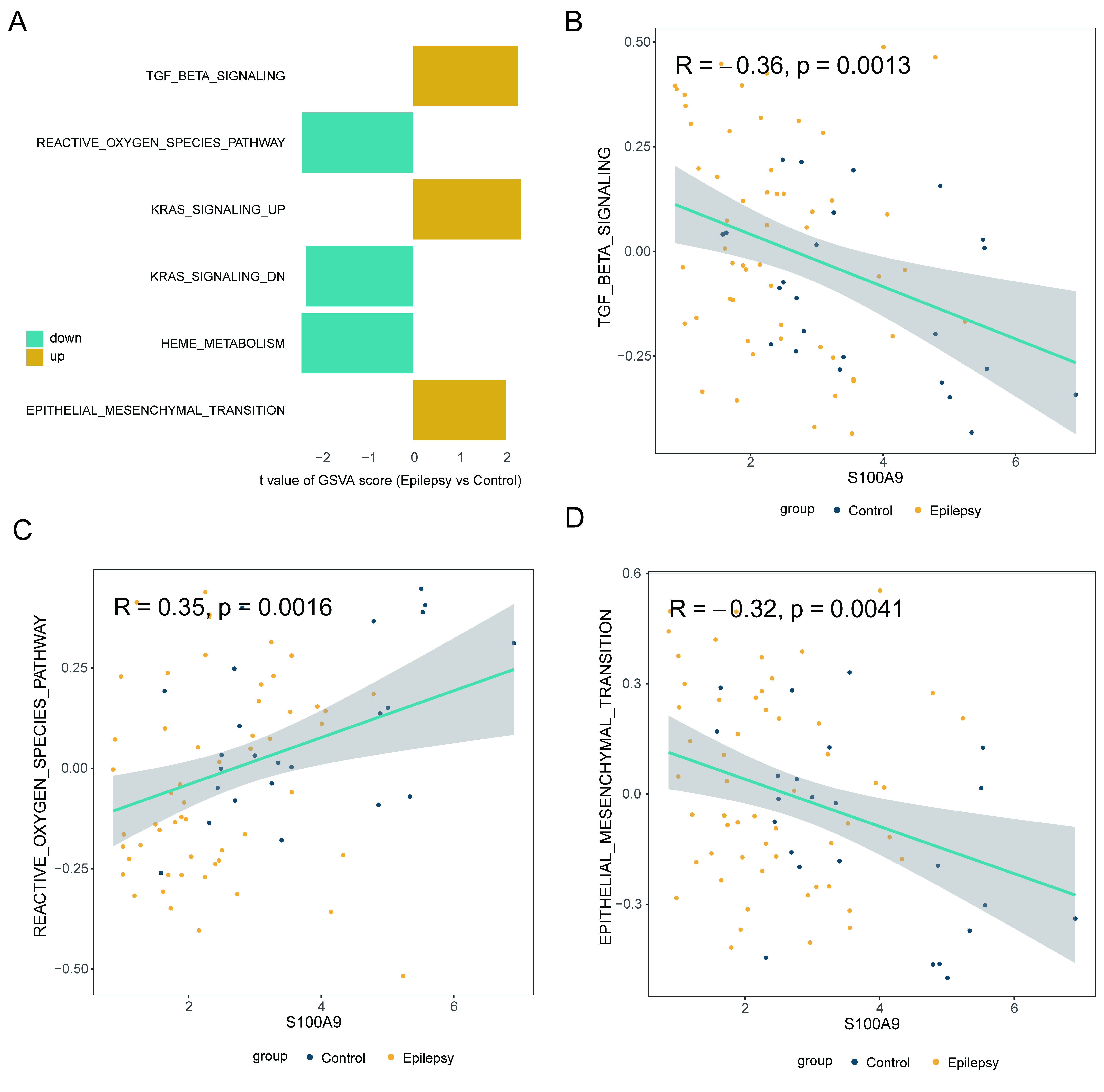

Supplement: Supplementary Figure 2 — GSVA results of epilepsy group and the control group. (A) Functional enrichment analysis in epilepsy group. (B) Spearman correlation between TGF_BETA_SIGNALING GSVA score and S100A9 expression. (C) Spearman correlation between REACTIVE_OXYGEN_SPECIES_PATHWAY GSVA score and S100A9 expression. (D) Spearman correlation between EPITHELIAL_MESENCHYMAL_TRANSITION GSVA score and S100A9 expression. “R” represents spearman correlation coefficient. [file Image_2.JPEG]

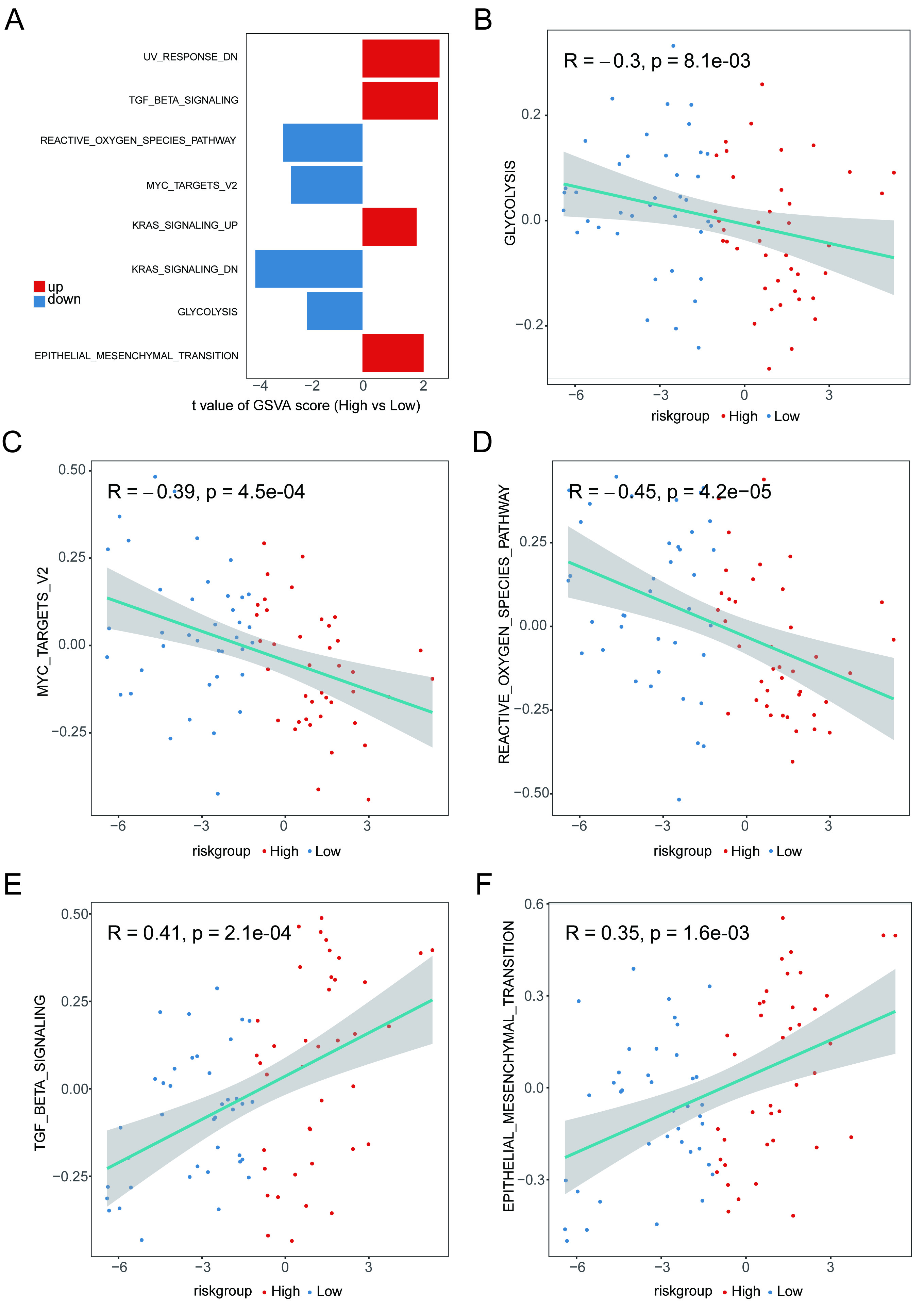

Supplement: Supplementary Figure 3 — GSVA analysis results for high and low-risk diagnostic groups. (A) Functional enrichment analysis in high and low-risk diagnostic groups. (B) Spearman correlation between GSVA score of GLYCOLYSIS pathway and risk diagnostic score. (C) Spearman correlation between GSVA score of MYC_TARGETS_V2 pathway and risk diagnostic score. (D) Spearman correlation between GSVA score of REACTIVE_OXYGEN_SPECIES_PATHWAY and risk diagnostic score. (E) Spearman correlation between GSVA score of TGF_BETA_SIGNALING pathway and risk diagnostic score. (F) Spearman correlation between GSVA score of EPITHELIAL_MESENCHYMAL_TRANSITION pathway and risk diagnostic score “R” represents spearman correlation coefficient. [file Image_3.JPEG]

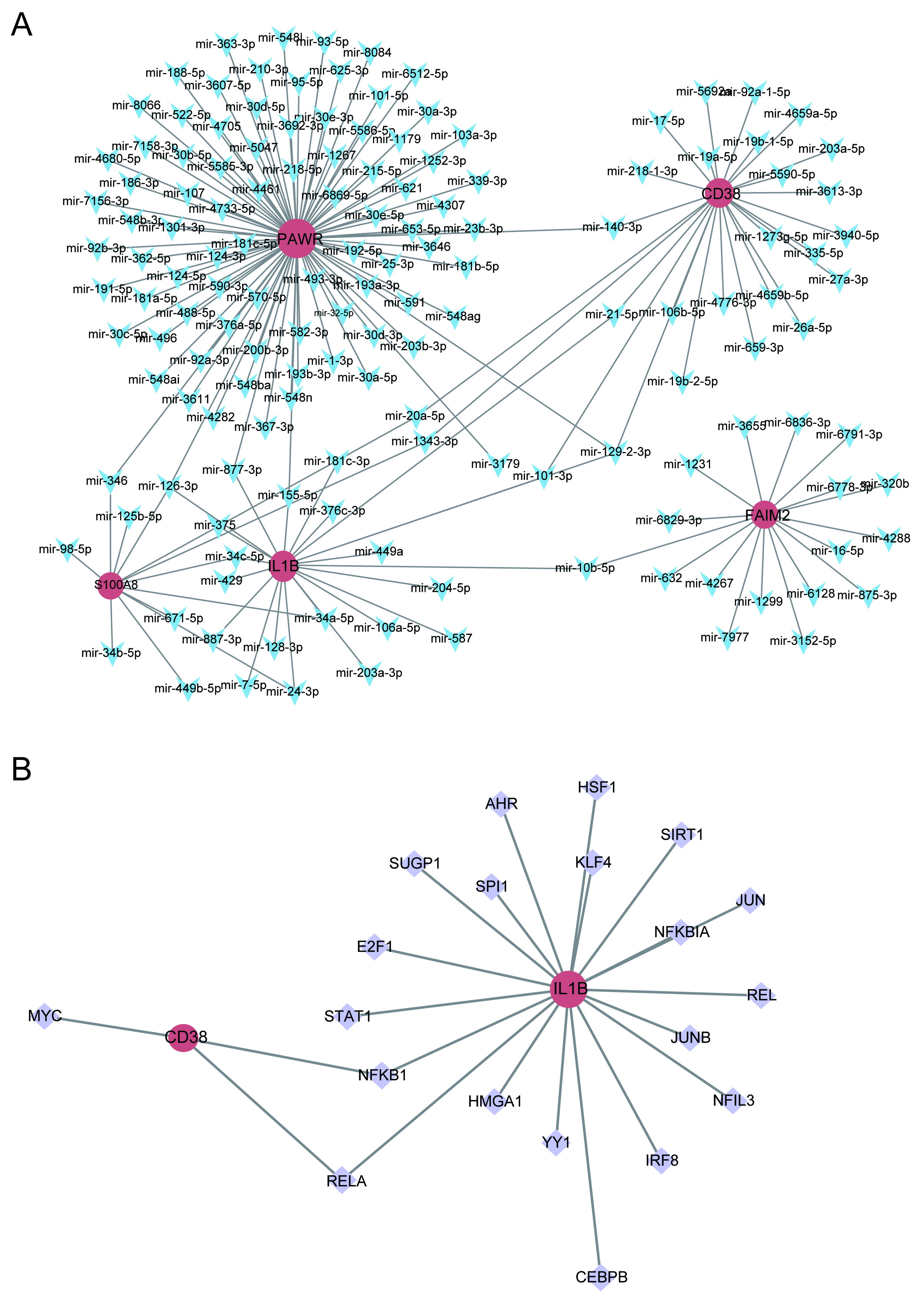

Supplement: Supplementary Figure 4 — Diagnostic gene and miRNA and TFs network. (A) Network of diagnostic genes and miRNAs. (B) Network of diagnostic genes and TFs. TF, transcription factor. [file Image_4.JPEG]

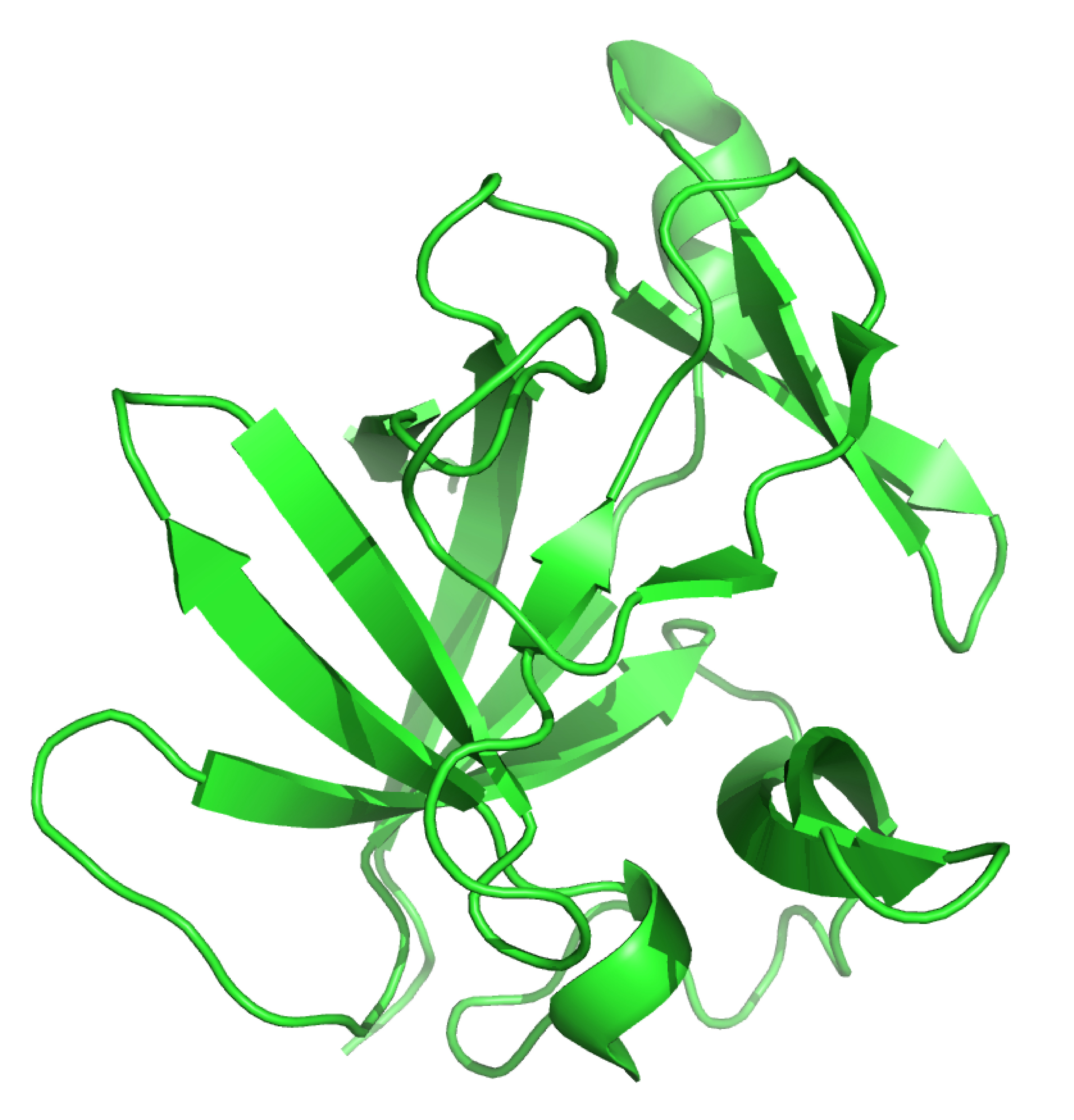

Supplement: Supplementary Figure 5 — Spatial structure of the 5R8Q configuration of IL1B. [file Image_5.JPEG]
